# Bioinformatic Identification and Expression Analysis of Banana MicroRNAs and Their Targets

**DOI:** 10.1371/journal.pone.0123083

**Published:** 2015-04-09

**Authors:** Juan Chai, Renjun Feng, Hourui Shi, Mengyun Ren, Yindong Zhang, Jingyi Wang

**Affiliations:** 1 Key Laboratory of Biology and Genetic Resources of Tropical Crops, Ministry of Agriculture, Institute of Tropical Bioscience and Biotechnology, Chinese Academy of Tropical Agricultural Sciences, Haikou, Hainan, China; 2 College of Agronomy, Hainan University, Haikou, Hainan, China; Mayo Clinic Arizona, UNITED STATES

## Abstract

MicroRNAs (miRNAs) represent a class of endogenous non-coding small RNAs that play important roles in multiple biological processes by degrading targeted mRNAs or repressing mRNA translation. Thousands of miRNAs have been identified in many plant species, whereas only a limited number of miRNAs have been predicted in *M*. *acuminata* (A genome) and *M*. *balbisiana* (B genome). Here, previously known plant miRNAs were BLASTed against the Expressed Sequence Tag (EST) and Genomic Survey Sequence (GSS), a database of banana genes. A total of 32 potential miRNAs belonging to 13 miRNAs families were detected using a range of filtering criteria. 244 miRNA:target pairs were subsequently predicted, most of which encode transcription factors or enzymes that participate in the regulation of development, growth, metabolism, and other physiological processes. In order to validate the predicted miRNAs and the mutual relationship between miRNAs and their target genes, qRT-PCR was applied to detect the tissue-specific expression levels of 12 putative miRNAs and 6 target genes in roots, leaves, flowers, and fruits. This study provides some important information about banana pre-miRNAs, mature miRNAs, and miRNA target genes and these findings can be applied to future research of miRNA functions.

## Introduction

MicroRNAs (miRNAs) are a class of endogenous non-coding RNA molecules that are 21–24 nucleotides (nt) in length. The processing and maturation of miRNAs in plants involves several steps that employ key enzymes, such as Dicer-like 1 (DCL1) [1] and HASTY [2,3]. Mature miRNAs are incorporated into the RNA-induced silencing complex (RISC) to post-transcriptionally regulate gene expression by targeting mRNA degradation or translation repression [4–6]. Previous research has shown that miRNAs play a key role in various metabolic and biological processes in plants, including meristem cell identity, leaf morphogenesis and polarity, floral differentiation and development, hormone signaling, organ separation, fruit growth and development, and the stress responses [7].

A number of methods have been applied to miRNA prediction, including genetic screening, direct cloning, sequencing methods, and computational approaches. Compared to these methods, the computational approach is one of the most effective, rapid, and affordable strategies to identify miRNAs [8] based on the major characteristic features of miRNAs, such as the hairpin-shaped stem-loop secondary structure with minimal folding free energy and high evolutionary conservation between species [9–11]. Using bioinformatics analysis based on comparative genome-based homolog searching, expressed sequence tag (EST) and/or genomic survey sequence (GSS), many researchers have identified miRNA genes from a variety of species, including *Arabidopsis thaliana* [12], *Oryza sativa* [13], *Triticum aestivum* [14], *Zea mays* [15], *Hordeum vulgare* [16], *Sorghum bicolor* [17], Gossypium [18], *Glycine max* [19], *solanum tuberosum* [20], *Lycopersicon esculentum* [21], citrus [22], *Fragaria vesca* [7], *Malus*. *domestica* [23], *Medicago truncatula* [24], *Brassica napus* [25], and *Catharanthus roseus* [26].

Bananas (*Musa* spp.) are giant perennial herbs from the order Zingiberales of the monocotyledon group, which originated in Southeast Asia and the western Pacific [27,28]. Cultivated bananas have evolved from the hybridization of wild species of *M*. *acuminata* and *M*. *balbisiana* [29]. These crops are of extreme importance across the world’s tropical and subtropical regions where they are vital for food security and are also a major source of incomes. Bananas are widely exported to industrialized countries where they constitute a major staple food for millions of people [27]. Bananas are the world’s most important fruit and rank fourth in the list of global food crops, with a global annual production in excess of 107 million tons [30].

Thousands of miRNAs belonging to various plant species have been registered in the miRNA database, miRBase [31] (http://www.mirbase.org), and novel miRNA genes will be identified in Release 20; however, none of these miRNAs are from *Musa*. Recently, a reference sequence of *Musa* A-genome and a draft sequence of B-genome have been published [27,32]. D’Hont et al (2012) reported 235 conserved miRNAs from 37 families using computational methods based on the‘Pahang’A-genome sequence similarity with known miRNAs in PMRD (http://bioinformatics.cau.edu.cn/PMRD/). Only the miR528 family out of 8 poales genomes-specific miRNA families was found in the A-genome. Davey et al (2013) predicted a similar number of microRNA sequences (47 and 42 miRNA families) in both A- and B-genomes, respectively, using publically available small RNA data from *M*.*acuminata*‘Calcutta 4’leaf, flower, and fruit tissues (sequenced within the framework of a NSF project http://smallrna.udel.edu) and 235 miRNA sequences reported for A-genome [27]. Davey et al (2013) also identified 28 novel miRNAs that were unique to either *Musa* A- or B-genomes, whereas, they did not experimentally verify and characterize the miRNAs.

In the present study, a computational analysis and a homolog search were used to identify banana miRNAs using miRBase, as well as EST and GSS data in National Center for Biotechnology Information (NCBI). The potential target genes of miRNAs were identified, and the potential functions of the predicted miRNAs targets, mainly the transcription factor genes, were investigated. Using real time fluorescent quantitative reverse transcription polymerase chain reaction (qRT-PCR), selected miRNAs and their targets were used to analyze the expression patterns in different tissues (i.e., roots, leaves, flowers, and fruits). The identification of miRNAs and their targets is important in order to understand the roles of miRNAs in the growth development and stress physiology of the banana (*Musa* spp.).

## Materials and Methods

### Plant material

Roots, leaves, flowers, and fruits were collected from banana plants (*Musa acuminata* L. AAA group, ‘Brazilian’) grown at the fruit science experimental station of Chinese Academy of Tropical Agricultural Sciences (19°98′N, 110°33′E; Haikou, Hainan province, China). After collection, all the samples were immediately frozen in liquid nitrogen and stored at -80°C until use.

### Sequences

A total of previously known plant miRNA sequences were obtained from miRBase [31] (http://www.mirbase.org/, Release 20.0, June 2013). In order to avoid overlap of sequences, repeats within the above sequences were removed. The remaining miRNA sequences were defined as a reference to find the conserved miRNAs in banana. A total of 46,111 banana EST and 31,544 GSS were obtained from the NCBI GenBank [33] in October 2013 (http://www.ncbi.nlm.nih.gov). These sequences were then used to predict the miRNAs and their target genes candidates.

### Prediction of miRNAs and their precursors

**Fig 1:**
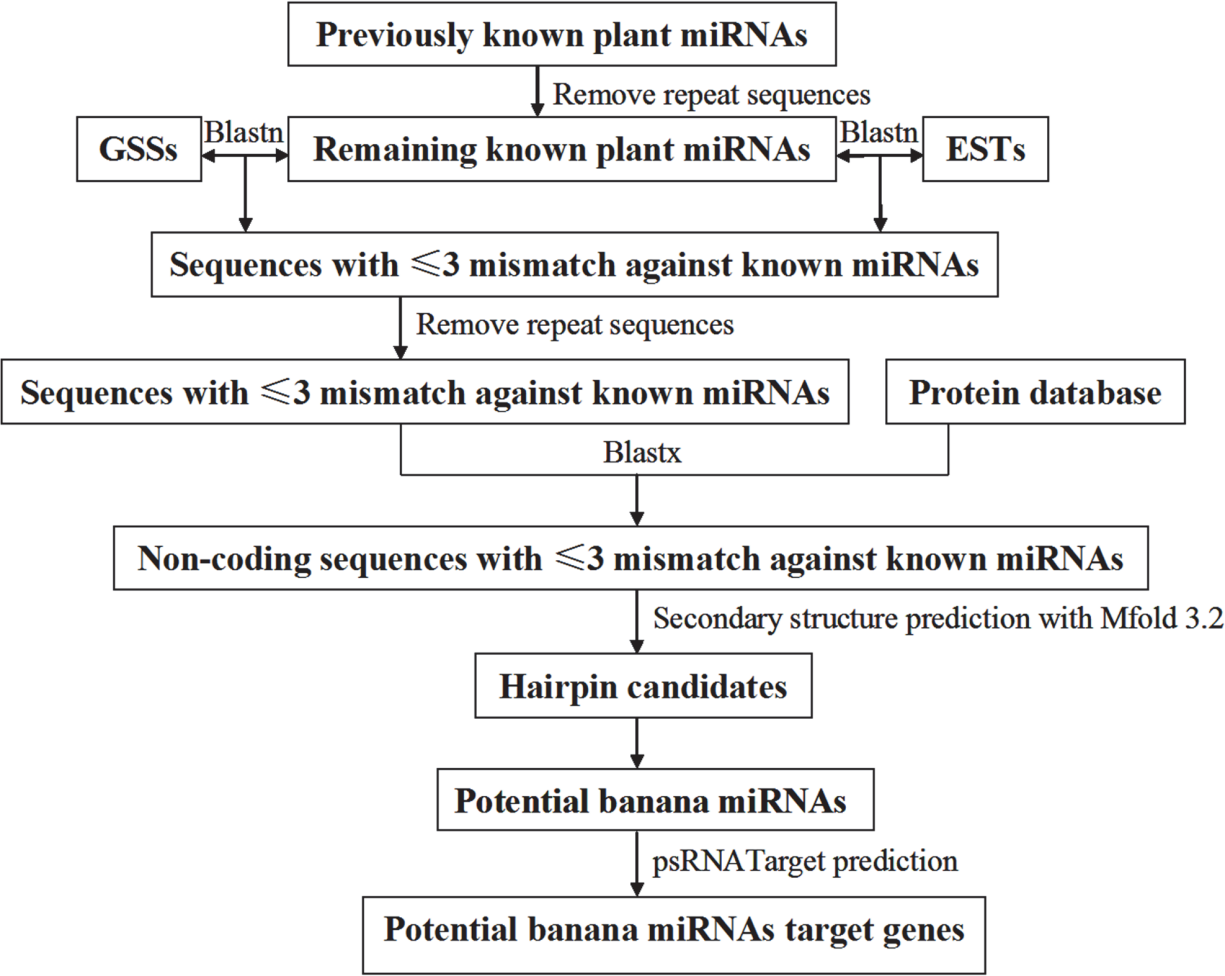
Flowchart of the banana miRNAs prediction procedure.

The prediction procedure is shown in Fig 1. First, known plant miRNAs in the miRBase were used as query sequences to blast against the downloaded ESTs and GSSs. The EST and GSS sequences that had no more than 3 mismatching nucleotides against the known miRNAs were regarded as the homologous sequences of the corresponding miRNAs, and the franking regions plus the matching nucleotide sequences were taken as the potential miRNA precursors. The repeat sequences and those coding proteins were then removed from the hit sequence using BLAST 2.2.14 (ftp://ftp.ncbi.nih.gov/blast/) and BLASTX (http://www.ncbi.nlm.nih.gov/BLAST/). Analysis of secondary structure and free energy of these sequences was conducted using the web-based software Mfold 3.2 [34] (http://mfold.rna.albany.edu/?q=mfold/RNA-Folding-Form).

These criteria were used for screening potential miRNAs or pre-miRNAs [35,36]: (1) predicted mature miRNAs have no more than 3 nt mismatches with the known miRNAs, (2) pre-miRNA sequence can fold into an appropriate hairpin secondary structure and the mature miRNA is located in one arm of the hairpin structure, (3) the mature miRNA sequence has less than 6 mismatches with the opposite miRNA* sequence in the other arm and no loop or break in miRNA/miRNA* duplex, (4) A+U content of pre-miRNA is ranged from 30% to 70%, and (5) the predicted secondary structure must have lower minimal folding free energy (MFE≦-20 kcal/mol).

### Prediction of the potential miRNA targets

Based on the newly identified potential miRNA sequences, we also predicted their potential targets using online software psRNATarget [37] (http://plantgrn.noble.org/psRNATarget/) with the following default parameters: maximum expectation of 3.0, length for complementarity scoring (hspsize) of 20 bp, target accessibility-allowed maximum energy to unpair the target site (UPE) of 25.0, flanking length around target site for target accessibility analysis of 17 bp in upstream and 13 bp in downstream, and range of central mismatch leading to translation inhibition of 9–11 nt.

### Expression analysis of banana miRNAs and target genes

Total RNA was isolated from different tissues following the CTAB method [38] and briefly exposed to RNAase-free DNAase I. cDNAs were synthesized by M-MLV according to the manufacturer’s instruction, using the specific stem-loop primers for miRNAs or Oligo(dT)15 for their target mRNAs. The specific stem-loop primers for miRNAs are listed in Table 1. After reverse transcription, the products of each reaction were diluted 10 times to avoid potential primer interference in the qRT-PCR reactions.

**Table 1:** Stem-loop RT primers and PCR primers for 12 identified miRNAs.

Name	Sequence(5’-3’)	
miR156d	stem-loop RT-primer	ctcaactggtgtcgtggagtccggcaattcagttgaggtgctctc
	PCR prime	Forward: acactccagctgggttgacagaag
miR156g	stem-loop RT-primer	ctcaactggtgtcgtggagtccggcaattcagttgaggtgctctc
	PCR prime	Forward: acactccagctgggtgacagaaga
miR166b	stem-loop RT-primer	ctcaactggtgtcgtggagtccggcaattcagttgagccccgagc
	PCR prime	Forward: acactccagctgggggaatgttgt
miR399a	stem-loop RT-primer	ctcaactggtgtcgtggagtccggcaattcagttgagcagggcaa
	PCR prime	Forward: acactccagctgggtgccaaagga
miR319m	stem-loop RT-primer	ctcaactggtgtcgtggagtccggcaattcagttgagagggagct
	PCR prime	Forward: acactccagctgggttggactgaa
miR160a	stem-loop RT-primer	ctcaactggtgtcgtggagtccggcaattcagttgagtggcatac
	PCR prime	Forward: acactccagctgggtgcctggctc
miR164e	stem-loop RT-primer	ctcaactggtgtcgtggagtccggcaattcagttgagtgcacgtg
	PCR prime	Forward: acactccagctgggtggagaagca
miR167c	stem-loop RT-primer	ctcaactggtgtcgtggagtccggcaattcagttgagcagatcat
	PCR prime	Forward: acactccagctgggtgaagctgcc
miR169h	stem-loop RT-primer	ctcaactggtgtcgtggagtccggcaattcagttgagcaggcaag
	PCR prime	Forward: acactccagctgggtagccaagga
miR162	stem-loop RT-primer	ctcaactggtgtcgtggagtccggcaattcagttgagtggacgca
	PCR prime	Forward: acactccagctgggtcgataaacc
miR5538	stem-loop RT-primer	ctcaactggtgtcgtggagtccggcaattcagttgaggcagcaag
	PCR prime	Forward: acactccagctgggactgaactca
miR4995	stem-loop RT-primer	ctcaactggtgtcgtggagtccggcaattcagttgagcccttaac
	PCR prime	Forward: acactccagctgggaggcagtggc
miR#	Universal reverse primer	Reverse: aactggtgtcgtggag
5S rRNA	PCR prime	Forward: tctggtgtcctaggcgta
		Reverse: attctggcgtcgagctat

#: 12 identified miRNAs.

The qRT-PCR reactions were performed using FastStart Universal SYBR Green Master (ROX) on a Mx3005P Real-Time PCR System. Briegly, 2.0 μL of diluted cDNA template was added to 12.5 μL of the FastStart Universal SYBR Green Master (ROX), 0.8 μM of each primer and ddH_2_O to a final volume of 25 μL. The reactions were amplified for 3 m at 95°C, followed by 35 or 40 cycles of 95°C for 10 s and 50°C for 30 s. A melting curve analysis was performed to determine the specificity of the products. Reactions were done in triplicate and the controls with no template and no reverse transcription were included for each gene. The banana 5S rRNA was used as an internal control. The forward and reverse primers used in real-time PCR are shown in Table 1 and Table 2.

**Table 2:** Target gene primers used for qRT-PCR.

Target gene	Sequence(5’-3’)
SPL 16 (GSMUA_Achr9T01380_001)	Forward: AAGTGCTTGGTGTATCCTC
	Reverse: GGCGTCGTCTTGTATGAA
SRPK4 (GSMUA_Achr8T04590_001)	Forward: AACCTTCTCACACTCATCAA
	Reverse: CACTGCCACCACAATCAT
WRKY19 (GSMUA_Achr8T27870_001)	Forward: GCTGACCAATGATGATGAAG
	Reverse: GTGTCGTGAATGTGTAACTC
GAMYB (GSMUA_Achr1T17150_001)	Forward: CATCAGTGGCAGTTCATTAG
	Reverse: TGGTGGTTCAGAAGGTAGA
DFR(GSMUA_Achr8T24070_001)	Forward: CCTGATGTAGTTGTTGATGAG
	Reverse: AGTATCTTCCTGATGCTGAG
F-box (GSMUA_Achr6T16010_001)	Forward: TTCTCCTCCTCTCCAAGTC
	Reverse: ACATCTCCTCCGCATTCT

The relative expression quantification was calculated using the equation 2−ΔΔCt, where ΔΔCt = (CtmiRNA—Ct5S rRNA)Tissue X—(CtmiRNA—Ct5S rRNA)Tissue 1[39]. In this study, tissue 1 presents the expression level of each miRNA in leaves, and the expression levels in root, flower and fruit tissues were quantified relative to this value. The whole assay was repeated three times in order to ensure the reliability of the assay data. The standard deviations of the data were obtained from three independent experiments. The 6 relative targets expression levels were also quantified using the 2−ΔΔCt method.

## Results

### Computational identification of banana miRNAs

The conservation of miRNA families among various plant species has facilitated the prediction of conserved miRNAs by EST and/or GSS analysis in various plants [36]. Using this method, we identified 32 conserved miRNAs derived from 16 EST and 16 GSS sequences, belonging to 13 miRNA families (Table 3). Eight and four predicted miRNA members belonged to the families of miR156/157 and miR166, respectively, while three members belonged to the family of, miR159/319, miR160, and miR162, respectively. The remaining miRNA families had no more than two members; among them, 28 miRNAs (87.5%) were derived from *M*. *acuminata* (AA and AAA) and 4 miRNAs (miR159, miR397, miR399a*, and miR399a) were derived from *Musa* ABB group; miR4995 and miR5538 are the new families in banana that have not been reported previously (Table 3).

**Table 3:** List of computer predicted banana miRNAs from the EST and GSS database.

Family	New miRNA	Mature sequence (5’-3’)	EST/GSS	Location	LM	LP	MFEs	A+U (%)
miR156/157	mac-miR156	GUUGACAGAAGAUAGAAGAGCAC	EST /JK538379	5’	22	112	-52.60	50.0
	mac-miR156a-5p	UGACAGAAGAGAGUGAGCAC	EST /ES434836	5’	20	94	-52.2	45.74
	mac-miR156a-3p	GCUCACUUCUCUUCCUGUCAGC	EST /ES434836	3’	22	94	-52.2	45.74
	mac-miR156d	UUGACAGAAGAUAGAGAGCAC	EST /JK538379	5’	21	112	-52.60	50.0
	mac-miR156g	UGACAGAAGAUAGAGAGCAC	GSS/HN242063	5’	20	140	-68.80	49.29
	mac-miR156h-3p	GCUCACUUCUCUUCCUGUCAGC	EST/ES434836	3’	22	94	-52.20	45.74
	mac-miR157b-5p	UUGACAGAAGAUAGAGAGCAC	GSS/HN242063	5’	21	140	-68.80	49.29
	mac-miR157b*	GCUCUCUAUGCUUCUGUCAUCA	GSS/HN242063	3’	22	140	-68.80	49.29
miR159/319	mbg-miR159	UUUGGAUUGAAGGGAGCUCUA	EST/FL661284	3’	21	129	-36.50	52.71
	mac-miR319c	UUGGACUGAAGGGAGCUCCC	GSS/HN244586	5’	20	108	-20.1	56.48
	mac-miR319m	UUGGACUGAAGGGAGCUCCCU	GSS/HN244586	5’	21	108	-20.1	56.48
miR160	mac-miR160a	UGCCUGGCUCCCUGUAUGCCA	EST/JK538087	3’	21	93	-20.9	50.54
	mac-miR160g-5p	UGCCUGGCUCCCUGUAUGCCA	GSS/HN248011	5	21	114	-49.30	47.37
	mac-miR160g*	GCGUGCAAGGAGCCAAGCAUG	GSS/HN248011	3’	21	114	-49.30	47.37
miR162	mac-miR162	UCGAUAAACCGCUGCGUCCA	GSS/HN247504	3’	20	144	-53.40	50.69
	mac-miR162*	GGAUGCAGAGGUUUAUCGACC	GSS/HN247504	3’	21	144	-53.40	50.69
	mac-miR162b	UCGAUAAACCGCUGCGUCCAG	GSS/HN247504	3’	21	144	-53.40	50.69
miR164	mac-miR164e	UGGAGAAGCAGGGCACGUGCA	GSS/HN246671	5’	21	176	-68.0	49.43
miR166	mac-miR166	UCGGACCAGGCUUCAUUCCCCC	EST/FF558732	3’	22	85	-43.1	43.53
	mac-miR166b	GGAAUGUUGUCUGGCUCGGGG	EST/FF558732	5’	21	85	-43.1	43.53
	mac-miR166c-5p	GGAAUGUUGUCUGGCUCGAGG	EST/FF558732	5’	21	85	-43.1	43.53
	mac-miR166c-3p	UCGGACCAGGCUUCAUUCCCC	EST/FF558732	3’	21	85	-43.1	43.53
miR167	mac-miR167c	UGAAGCUGCCAGCAUGAUCUG	GSS/HN250108	3’	21	149	-31.70	56.38
	mac-miR167d	UGAAGCUGCCAGCAUGAUCUGG	GSS/HN250108	3’	22	149	-31.70	56.38
miR169	mac-miR169h	UAGCCAAGGAUGACUUGCCUG	EST/JK539556	5’	21	103	-52.2	44.66
miR172	mac-miR172b	UGAAUCUUAAUGAUGCUACA	GSS/HN239201	3’	20	123	-42.80	56.91
	mac-miR172c	UGAAUCUUAAUGAUGCUACAC	GSS/HN239201	3’	21	123	-42.80	56.91
miR397	mbg-miR397a	UCAUUGAGUGCAGCGUUGAUG	EST/FL666615	5’	21	125	-58.50	49.60
miR399	mbg-miR399a	GUGCAGUUCUCCUCUUGGCAA	EST/FL666459	5’	21	92	-48.6	45.65
	mbg-miR399a*	UGCCAAAGGAGAAUUGCCCUG	EST/FL666459	3’	21	92	-48.6	45.65
miR4995	mac-miR4995	AGGCAGUGGCUUGGUUAAGGG	EST/DN238208	3’	21	77	-21.80	44.16
miR5538	mac-miR5538	ACUGAACUCAAUCACUUGCUGC	GSS/DX457639	3’	22	131	-19.1	58.78

Note: mac:Musa acuminata; mbg: Musa ABB Group The underlined was the mismatched bases between miRNAs and the known miRNA sequences in the miRBase; Location: the position of mature miRNA in precursor; LM: length of mature miRNA (nt); LP: length of precursor (nt); MFEs: minimal folding free energies (kcal mol^−1^). *: star miRNA.

Though the lengths of miRNA precursors varied from 77 nt to 176 nt (Table 3), the lengths of mature miRNAs were mainly confined to 21 nucleotides (65.6% = 21/32), except five (15.6% = 5/32) (miR156a-5p, miR156g, miR319c, miR162, and miR172b) and seven (21.9% = 7/32) miRNAs (miR156, miR156a-3p, miR156h, miR157b*, miR166, miR167d, and miR5538) with 20 and 22 nucleotides, respectively. The A+U contents of the pre-miRNAs ranged from 43.53% to 58.78%, with an average of 49.32%, which met the previous criteria. Mature miRNA sequences have been reported to be evenly located on the 2 arms of the stem-loop hairpin structures of potential pre-miRNAs [40]. In our study, slightly more mature miRNAs were located in the 3′end of the pre-miRNA than were located in the 5′end (17:15). In addition, 5 pairs of miRNA/miRNA* were located at the same precursors. Among the miRNAs, 65.6% (21/32) of members begin with a 5′uracil. These results are in agreement with previous observations [19; 41,42]. All 33 miRNAs had fewer than six mismatched base pairs between the region of the mature miRNA sequences and its opposite arms. MFE is an important parameter in determining the stability of the perfect or near-perfect secondary hairpin structure of pre-miRNA, as a lower MFE value generally indicates a more stable secondary structure. In the present study, the average value of MFE was -45.3 kcal/mol with a range of -19.1 kcal/mol to -68.8 kcal/mol.

### Conservation of the newly identified banana miRNAs

**Fig 2:**
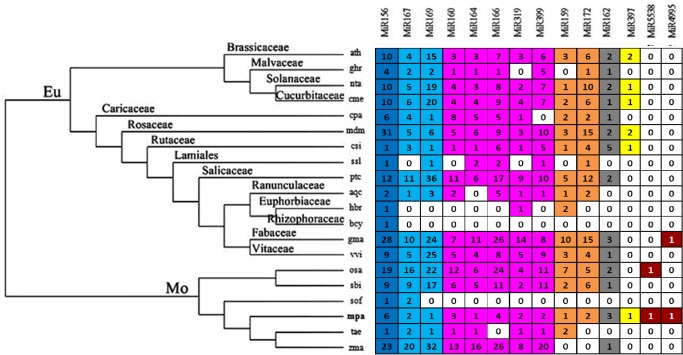
The statistical of the miRNAs distribution in different plants. Digital number on behalf of the members of the family. The species names are abbreviated as following: Eu, *Eudicotyledons*; Mo, *Monocotyledons*; ath, *Arabidopsis thaliana*; ghr, *Gossypium hirsutum*; nta, *Nicotiana tabacum*; cme, *Cucumis melo*; cpa, *Carica papaya*; mdm, *Malus domestica*; csi, *Citrus sinensis*; ssl, *Salvia sclarea*; ptc, *Populus trichocarpa*; aqc, *Aquilegia caerulea*; hbr, *Hevea brasiliensis*; bcy, *Bruguiera cylindrica*; gma, *Glycine max*; vvi, *Vitis vinifera*; osa, *Oryza sativa*; sbi, *Sorghum bicolor*; sof, *Saccharum officinarum*; mpa, *Musa paradisiaca*; tae, *Triticum aestivum*; zma, *Zea mays*.

The miRNA families found in the miRBase are classified as conserved or unique depending on the number of species in which they have been identified. miRNA families registered in single species are considered to be unique, and those registered in two species and three or more species as moderately and highly conserved, respectively [43]. We analyzed 14 miRNAs registered in miRBase in 19 species. Multiple sequence alignment for 14 miRNAs registered in miRBase in19 species have shown in S1 File. Based on our results (Fig 2), 11 of the 13 miRNAs families are conserved within the vegetable kingdom, mainly *Arabidopsis thalinana*, *Nicotiana tabacum*, *Malus domestica*, *Cucumis melo*, *Glycine max*, *Oryza sativa*, and *Zea mays*, but the miR4995 and miR5538 family are exceptions. miR4995 and miR5538 show conservation with that of *Glycine max* and *Oryza sativa* respectively.

### Prediction of banana miRNA target genes

Based on the complementarity between miRNAs and their target genes in plants, all gene-coding sequences in the banana genome database (http://banana-genome.cirad.fr/download/musa_cds.fna.gz) were used to predict banana miRNA targets by psRNATarget (http://plantgrn.noble.org/psRNATarget/). 244 miRNA:target pairs between 32 miRNAs and 121 mRNAs have been predicted.

Table 4 shows some potential target-genes harboring binging sites for 13 conserved miRNA families. A complete list of predicted targets is depicted in S1 Table. Except for miR5538, every miRNA has more than one target. Further analysis found that most of the highly conserved miRNA families target transcription factors, such as the SBP family targeted by miR156, TCP and MYB families targeted by miR159/319, ARF family targeted by miR160, NAC family targeted by miR164, HD-Zip family targeted by miR166, and WRKY family targeted by miR399 (Table 4). In addition, other predicted target genes are involved in diverse metabolic and physiological processes, such as dehydrogenase, decarboxylase, helicase, synthase, protein kinases, oxidoreductase, binding protein, lipase, transporters, disease resistance protein, f-box proteins, zinc finger protein, heat shock protein, and laccase (Table 4).

**Table 4:** Putative target genes of miRNA candidates identified in banana.

miRNA family	Target ptotein	Function
miR156/157	Squamosa promoter-binding-like protein	Transcription factor
	OsSPL16—SBP-box gene family member	Transcription factor
	ELMO/CED-12 family protein	Signal transduction
	CTP synthase 1	Metabolism
	Putative Abscisic acid 8'-hydroxylase 3	Metabolism/Signal transduction
	RNA recognition motif containing protein	Metabolism
miR159/319	Transcription factor GAMYB	Transcription factor
	TCP family transcription factor	Transcription factor
	Chorismate mutase, chloroplastic	Metabolism
	Putative Nuclear polyadenylated RNA-binding protein 4	Transcription factor
	Putative Cysteine-rich receptor-like protein kinase 15	Signal transduction
miR160	Auxin response factor	Transcription factor
	pentatricopeptide repeat-containing protein At1g68930	Metabolism
miR162	Anthranilate synthase component I-1, chloroplastic	Growth development
	Nicotianamine synthase 3	Metabolism
miR164	NAC domain-containing protein 21/22 (NAC21/22/NAM/CUC2)	Transcription factor
	UDP-glucuronic acid decarboxylase 1	Transcriptional regulation
	E3 ubiquitin-protein ligase BRE1-like 1	Signal transduction
	disease resistance protein, putative, expressed	Stress responses
miR166	HD-ZIPⅢ	Transcription factor
	SRPK4	Transcription factor
	Putative 2-oxoglutarate dehydrogenase, mitochondrial	Metabolism
	pectinacetylesterase domain containing protein, expressed	Metabolism
	Putative U-box domain-containing protein 33	Signal transduction/protein metabolism
	GDSL esterase/lipase At5g33370	Metabolism
miR167	Putative tRNA-specific adenosine deaminase 1	Metabolism
	Putative U-box domain-containing protein 52	Signal transduction/protein metabolism
	Cytochrome c biogenesis protein CCS1, chloroplastic	Metabolism
miR169	Glutamate synthase [NADH], amyloplastic	Metabolism
miR172	amine oxidase-related	Metabolism
miR397	Laccase	Stress response
	Putative pectinesterase/pectinesterase inhibitor 28	Metabolism
	Putative S-(hydroxymethyl)glutathione dehydrogenase	Metabolism/oxidative stress response
	Putative Heat shock 70 kDa protein 4L	Stress response
	cbbY protein-related, putative, expressed	Metabolism
miR399	Probable WRKY transcription factor 19	Transcription factor
	Putative Zinc finger protein NUTCRACKER	Transcription factor
	2-oxoglutarate/malate translocator, chloroplastic	Transport
	DEAD-box ATP-dependent RNA helicase 37	Metabolism
	Putative Exportin-2	Transport
miR4995	F-box protein At2g32560	Transcription factor
	zinc knuckle domain containing protein	Metabolism
	Bifunctional aspartokinase/homoserine dehydrogenase 1	Metabolism
miR5538	Dihydroflavonol-4-reductase	Metabolism

### miRNAs and target confirmation by RT-qPCR

**Fig 3:**
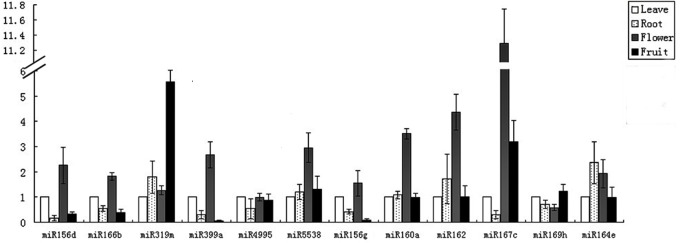
The relative expression level of selected miRNAs in leave, root, flower, and fruit of banana. The horizontal axis indicates the 12 miRNAs, and the vertical axis indicates the relative expression.

In order to examine the expression of these miRNAs, stem-loop RT-PCR were performed using a total RNA isolated from roots, leaves, flowers, and fruits. Using this approach, we were able to detect a positive signal of the expected size at 70nt (data not shown), and to confirm the presence of these miRNAs. 12 miRNAs from 11 families were chosen randomly in this experiment. All the PCR products were cloned and verified by sequencing. 12 potential miRNAs were expressed and exhibited different expression patterns in four tissues (Fig 3). The expression levels of miR164e, miR169h, and miR4995 were not significantly different among these four tissues. MiR156d, miR166b, miR399a, miR5538, miR156g, miR160a, miR162, and miR167c presented similar orders of expression in leaves, roots, flowers and fruits, having the highest expression levels in the flower tissue. When the expression levels of tested miRNAs in different tissues were compared, miR167c, miR319m, and miR164e had the highest expression level in flowers, fruits, and roots respectively (11.26, 5.62, and 2.50 folds of that in leave tissues, respectively), whereas miR399a, miR156d, and miR169h represented the lowest expression level in fruits, roots, and flowers, respectively; thus, the conserved miRNAs were expressed in a tissue-specific manner.

**Fig 4:**
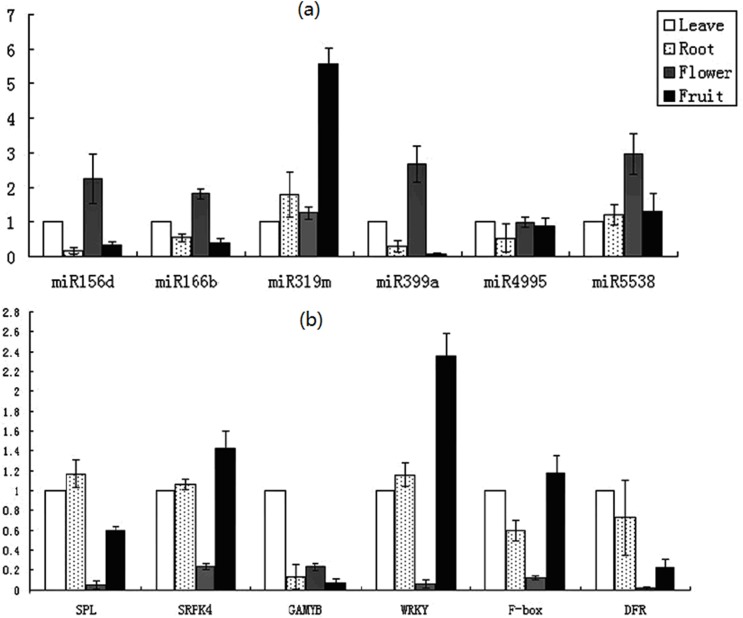
The relative expression level of selected six miRNAs (a) and their targets (b) in leave, root, flower, and fruit of banana. The horizontal axis indicates the 6 miRNAs and their targets, and the vertical axis indicates the relative expression.

In order to compare the expression of targets with that of the miRNAs, qRT-PCR was also used to confirm the expression of computationally predicted banana miRNA target genes in leaves, roots, flowers, and fruits; these miRNA target genes included *SPL16*, *SRPK4*, *GAMYB*, *WRKY19*, *F-box*, and *DFR*. The expression patterns of target genes in banana tissues were negatively correlated with the miRNA quantification results (Fig 4): the lowest expression levels of the miR156d, miR166b, miR399a, miR4995, and miR5538 target genes were consistent with the highest expression levels of miR156d, miR166b, miR399a, miR4995, and miR5538 in the flower tissue. The lowest expression level of the miR319 target gene was consistent with the highest expression level of miR319 in the fruit tissue. The higher expression levels of the six target genes were consistent with the lower expression of the six miRNAs in four tissues.

## Discussion

Although miRNAs have been extensively studied for several years, no systematic study has been performed on the banana, one of the most important subtropical crops in the world. Recently, D’Hont et al (2012) and Davey et al (2013) identified 37 miRNA families and 42 miRNA families from *Musa* A- and B-genome, respectively, using bioinformatics analysis: those predicted miRNAs have not been confirmed by experiments for their precise sequences. Thus, banana miRNAs still remain largely unknown. Though there are a few computational approaches to identify miRNAs of plants, animals and even microorganisms, EST/GSS analysis has some advantages over other methods [19,44].

Using EST and GSS analysis, we identified 32 miRNAs belonging to 13 miRNA families. Among them, miR4995 and miR5538 are the newly found families in banana that have not previously been reported. The frequency of miRNA from ESTs and GSSs was 0.037% and 0.051%, respectively, which is little higher than the rate of 0.01% (1 in 10,000 ESTs) reported by Zhang et al (2006). 11 miRNA families identified in the study were the same as previously reported results [27,32], which have, to some extent, verified the utility of the computational predication of potential miRNAs. Several studies showed that many miRNAs are conserved across different plants [1]; however, some miRNAs are species-specific. For instance, miR5538 family is present in rice but not in *Arabidopsis*, suggesting that it might be restricted to monocots [45]. Cloning and sequencing validated 12 miRNAs belonging to 11 families; moreover, the ubiquitous expression of these 12 miRNAs could be detected in different banana tissues ranging from roots, leaves, flowers and fruits by qRT-PCR and some showed differential expression in different tissues. Our qRT-PCR analysis validated the miRNA prediction in banana, and their preferential expression can provide important clues about the miRNAs’ function.

The prediction of miRNA target genes is important for understanding miRNA gene-regulation. Currently, bioinformatics methods based on the high homology between miRNAs and target genes are considered one of the most efficient approaches used to verify target genes, and has been employed in numerous studies [22; 46–49]. Using the newly identified miRNAs in banana as query sequences to blast against the banana genome database, we were able to predict various miRNA target genes involved in transcription, translation, stress response, structural component, development, and metabolism in the banana (Table 4).

We predicted 244 miRNA:target pairs for 32 miRNAs giving an average miRNA to target ratio of 7.6 (244/32), which is greater than the ratios of 2.8 for the apple [23] and 4.1 for the castor bean [48]. Consistent with previous finding, most of these targets in the banana were plant-specific transcription factors, such as SBP, TCP, and MYB, NAC, ARF, and the HD-ZIP family, which were also predicted as targets of miR156/157, miR159/319, miR164, miR160 and miR166 in Arabidopsis [46,50], tobacco [42], citrus [22], apples [23], castor beans [48], and papayas [49]. MiR397 family targets Laccase were also conserved among plants; however, the other three miRNA families target genes that are found in a broad range of plants were not predicted to target genes with similar functions in the current banana database: miR162:DCL1 (Dicer1), miR167:ARF (auxin response factor), and miR172:AP2 (*APETALA2*).

qRT-PCR was used to confirm the expression of potential banana miRNAs and their targets. The expression patterns between miRNAs and their targets showed a negative correlation [51,52]. In our study, the lower expression levels of the six target genes were consistent with the higher expression of the six miRNAs in four tissues, indicating that they may be actively cleaved by miRNAs (Fig 4). Squamosa promoter-binding protein-like (*SPL*) is the target of miR156 family. *SPL* genes encode plant-specific transcription factors that play important roles in fruit ripening [53], ovary and fruit development [54], grain size, shape, and quality [55], plant phase transition, plant architecture, and gibberellins signaling [56]. Our study showed that the decrease of miR156d expression may lead to increased expression of the *SPL16* in roots and fruits in the banana. MiR319 plays an important role in flower organ identity and cold-induced stress [51,57]. The lowest expression level of the miR319m target gene (*GAMYB*) was consistent with the highest expression level of miR319m in the banana fruits. *GAMYB* has been demonstrated to play an important role in flower development, especially in stamen and anther development [58–60]; furthermore, *GAMYB* is also involved in GA-mediated programmed cell death (PCD) in the aleurone and tapetum [61].

MiR5538 showed the highest expression in flowers, and the target of miR5538, *DFR* (Dihydroflavonol-4-reductase), one of the key enzymes involved in flavonoids biosynthetic pathway, was expressed at the lowest level in the banana flowers. Flavonoids are involved in several biological processes including pigmentation of flowers, seed development, pollen viability, auxin transport regulation, protection against UV-B injury, defense against pathogens, and pests [62–64]; this may indicate that miR5538 has an important role in flower development and pollen viability in banana. F-box proteins, as targets for miR393/394, are reported to be differentially regulated by stress conditions and to play important roles in the abiotic stress response pathway [10,65]. In our study, miR4995 targets F-box protein (At2g32560), zinc knuckle domain containing protein, and bifunctional aspartokinase/homoserine dehydrogenase 1. The function of miR4995 is still not clear. Further studies will need to be performed in order to determine the regulation pattern of banana miRNAs and their targets.

## Supporting Information

S1 File Multiple sequence alignment for 14 miRNAs registered in miRBase in19 species. (DOC)

S1 Table Putative target genes of miRNA candidates identified in banana. (XLS)
