# Supplementary material for: Bioinformatic Identification and Expression Analysis of Banana MicroRNAs and Their Targets
Source: PLoS One. 2015 Apr 9;10(4):e0123083. doi: 10.1371/journal.pone.0123083 (PMC4391839; doi:10.1371/journal.pone.0123083)
Supplement: S1 File — (DOC) [file pone.0123083.s001.doc]

File S1. Multiple sequence alignment for 14 miRNAs registered in miRBase in19 species. The species names are abbreviated as following: Eu, *Eudicotyledons*; Mo, *Monocotyledons*; ath, *Arabidopsis thaliana*; ghr, *Gossypium hirsutum*; nta, *Nicotiana tabacum*; cme, *Cucumis melo*; cpa, *Carica papaya*; mdm, *Malus domestica*; csi*, Citrus sinensis*; ssl, *Salvia sclarea*; ptc, *Populus trichocarpa*; aqc, *Aquilegia caerulea*; hbr, *Hevea brasiliensis*; bcy, *Bruguiera cylindrica*; gma, *Glycine max*; vvi, *Vitis vinifera*; osa, *Oryza sativa*; sbi, *Sorghum bicolor*; sof, *Saccharum officinarum*; tae, *Triticum aestivum*; zma, *Zea mays.* The miRNAs identified in the study are in red color.

1.miR156

sbi-miR156f ---UGACAGAAGAGA--GUGAGCAC-- 20

sbi-miR156c ---UGACAGAAGAGA--GUGAGCAC-- 20

sbi-miR156b ---UGACAGAAGAGA--GUGAGCAC-- 20

sbi-miR156g ---UGACAGAAGAGA--GUGAGCAC-- 20

sbi-miR156a ---UGACAGAAGAGA--GUGAGCAC-- 20

osa-miR156b-5p ---UGACAGAAGAGA--GUGAGCAC-- 20

osa-miR156j-5p ---UGACAGAAGAGA--GUGAGCAC-- 20

osa-miR156e ---UGACAGAAGAGA--GUGAGCAC-- 20

osa-miR156i ---UGACAGAAGAGA--GUGAGCAC-- 20

cme-miR156j -GUUGACAGAAGAGA--GUGAGCAC-- 22

cme-miR156a ---UGACAGAAGAGA--GUGAGCAC-- 20

cpa-miR156a ---UGACAGAAGAGA--GUGAGCAC-- 20

cpa-miR156d ---UGACAGAAGAGA--GUGAGCAC-- 20

sof-miR156 ---UGACAGAAGAGA--GUGAGCAC-- 20

zma-miR156c ---UGACAGAAGAGA--GUGAGCAC-- 20

zma-miR156h-5p ---UGACAGAAGAGA--GUGAGCAC-- 20

mdm-miR156k ---UGACAGAAGAGA--GUGAGCAC-- 20

mdm-miR156e ---UGACAGAAGAGA--GUGAGCAC-- 20

mdm-miR156i ---UGACAGAAGAGA--GUGAGCAC-- 20

mdm-miR156n ---UGACAGAAGAGA--GUGAGCAC-- 20

mdm-miR156f ---UGACAGAAGAGA--GUGAGCAC-- 20

ptc-miR156a ---UGACAGAAGAGA--GUGAGCAC-- 20

ptc-miR156c ---UGACAGAAGAGA--GUGAGCAC-- 20

gma-miR156a ---UGACAGAAGAGA--GUGAGCAC-- 20

gma-miR156v ---UGACAGAAGAGA--GUGAGCAC-- 20

zma-miR156f-5p ---UGACAGAAGAGA--GUGAGCAC-- 20

gma-miR156x ---UGACAGAAGAGA--GUGAGCAC-- 20

vvi-miR156c ---UGACAGAAGAGA--GUGAGCAC-- 20

osa-miR156c-5p ---UGACAGAAGAGA--GUGAGCAC-- 20

osa-miR156g-5p ---UGACAGAAGAGA--GUGAGCAC-- 20

gma-miR156s ---UGACAGAAGAGA--GUGAGCACU- 21

gma-miR156q ---UGACAGAAGAGA--GUGAGCACU- 21

ath-miR156b-5p ---UGACAGAAGAGA--GUGAGCAC-- 20

ath-miR156c-5p ---UGACAGAAGAGA--GUGAGCAC-- 20

ath-miR156a-5p ---UGACAGAAGAGA--GUGAGCAC-- 20

ghr-miR156a ---UGACAGAAGAGA--GUGAGCAC-- 20

ghr-miR156b ---UGACAGAAGAGA--GUGAGCAC-- 20

ath-miR156g ---CGACAGAAGAGA--GUGAGCAC-- 20

osa-miR156l-5p ---CGACAGAAGAGA--GUGAGCAUA- 21

ssl-miR156 ---UGACAGAAGAGA--GUGAGCACA- 21

tae-miR156 ---UGACAGAAGAGA--GUGAGCACA- 21

ath-miR156f-5p ---UGACAGAAGAGA--GUGAGCAC-- 20

ath-miR156e ---UGACAGAAGAGA--GUGAGCAC-- 20

ath-miR156d-5p ---UGACAGAAGAGA--GUGAGCAC-- 20

ghr-miR156d ---UGACAGAAGAGA--GUGAGCAC-- 20

nta-miR156a ---UGACAGAAGAGA--GUGAGCAC-- 20

nta-miR156c ---UGACAGAAGAGA--GUGAGCAC-- 20

nta-miR156d ---UGACAGAAGAGA--GUGAGCAC-- 20

nta-miR156b ---UGACAGAAGAGA--GUGAGCAC-- 20

sbi-miR156i ---UGACAGAAGAGA--GUGAGCAC-- 20

nta-miR156e ---UGACAGAAGAGA--GUGAGCAC-- 20

cme-miR156c ---UGACAGAAGAGA--GUGAGCAC-- 20

cme-miR156i ---UGACAGAAGAGA--GUGAGCAC-- 20

cme-miR156d ---UGACAGAAGAGA--GUGAGCAC-- 20

cpa-miR156c ---UGACAGAAGAGA--GUGAGCAC-- 20

sbi-miR156h ---UGACAGAAGAGA--GUGAGCAC-- 20

zma-miR156l-5p ---UGACAGAAGAGA--GUGAGCAC-- 20

zma-miR156e-5p ---UGACAGAAGAGA--GUGAGCAC-- 20

zma-miR156b-5p ---UGACAGAAGAGA--GUGAGCAC-- 20

mdm-miR156a ---UGACAGAAGAGA--GUGAGCAC-- 20

mdm-miR156b ---UGACAGAAGAGA--GUGAGCAC-- 20

mdm-miR156o ---UGACAGAAGAGA--GUGAGCAC-- 20

mdm-miR156m ---UGACAGAAGAGA--GUGAGCAC-- 20

csi-miR156 ---UGACAGAAGAGA--GUGAGCAC-- 20

ptc-miR156f ---UGACAGAAGAGA--GUGAGCAC-- 20

ptc-miR156b ---UGACAGAAGAGA--GUGAGCAC-- 20

gma-miR156y ---UGACAGAAGAGA--GUGAGCAC-- 20

zma-miR156a-5p ---UGACAGAAGAGA--GUGAGCAC-- 20

gma-miR156h ---UGACAGAAGAGA--GUGAGCAC-- 20

vvi-miR156d ---UGACAGAAGAGA--GUGAGCAC-- 20

osa-miR156a ---UGACAGAAGAGA--GUGAGCAC-- 20

osa-miR156h-5p ---UGACAGAAGAGA--GUGAGCAC-- 20

osa-miR156d ---UGACAGAAGAGA--GUGAGCAC-- 20

osa-miR156f-5p ---UGACAGAAGAGA--GUGAGCAC-- 20

vvi-miR156b ---UGACAGAAGAGA--GUGAGCAC-- 20

mac-miR156a-5p ---UGACAGAAGAGA--GUGAGCAC-- 20

gma-miR156w ---UGACAGAAGAGA--GUGAGCAC-- 20

gma-miR156u ---UGACAGAAGAGA--GUGAGCAC-- 20

ptc-miR156d ---UGACAGAAGAGA--GUGAGCAC-- 20

ptc-miR156e ---UGACAGAAGAGA--GUGAGCAC-- 20

mdm-miR156c ---UGACAGAAGAGA--GUGAGCAC-- 20

mdm-miR156l ---UGACAGAAGAGA--GUGAGCAC-- 20

mdm-miR156h ---UGACAGAAGAGA--GUGAGCAC-- 20

mdm-miR156g ---UGACAGAAGAGA--GUGAGCAC-- 20

mdm-miR156j ---UGACAGAAGAGA--GUGAGCAC-- 20

zma-miR156i-5p ---UGACAGAAGAGA--GUGAGCAC-- 20

zma-miR156g-5p ---UGACAGAAGAGA--GUGAGCAC-- 20

zma-miR156d-5p ---UGACAGAAGAGA--GUGAGCAC-- 20

mdm-miR156d ---UGACAGAAGAGA--GUGAGCAC-- 20

cpa-miR156b ---UGACAGAAGAGA--GUGAGCAC-- 20

vvi-miR156e ---UGACAGAGGAGA--GUGAGCAC-- 20

gma-miR156o --UUGACAGAAGAGA--GUGAGCAC-- 21

gma-miR156n --UUGACAGAAGAGA--GUGAGCAC-- 21

gma-miR156k --UUGACAGAAGAGA--GUGAGCAC-- 21

nta-miR156f ---UGACAGAAGAGA--AUGAGCAC-- 20

ghr-miR156c ---UGUCAGAAGAGA--GUGAGCAC-- 20

mdm-miR156ad ---UGACAGAAGAAA--GUGAGCAC-- 20

mdm-miR156ae ---UGACAGAAGAAA--GUGAGCAC-- 20

sbi-miR156e ---UGACAGAAGAGA--GCGAGCAC-- 20

zma-miR156k-5p ---UGACAGAAGAGA--GCGAGCAC-- 20

cme-miR156g ---UGACAGAAGAGA--GGGAGCAC-- 20

cme-miR156h ---UGACAGAAGAGA--GGGAGCAC-- 20

ptc-miR156k ---UGACAGAAGAGA--GGGAGCAC-- 20

vvi-miR156a ---UGACAGAAGAGA--GGGAGCAC-- 20

gma-miR156b ---UGACAGAAGAGA--GAGAGCACA- 21

osa-miR156k ---UGACAGAAGAGA--GAGAGCACA- 21

sbi-miR156d ---UGACAGAAGAGA--GAGAGCACA- 21

zma-miR156j-5p ---UGACAGAAGAGA--GAGAGCACA- 21

gma-miR156f --UUGACAGAAGAGA--GAGAGCACA- 22

ath-miR156j ---UGACAGAAGAGA--GAGAGCAC-- 20

ath-miR156i ---UGACAGAAGAGA--GAGAGCAG-- 20

vvi-miR156h ---UGACAGAAGAGA--GAGAGCAU-- 20

mdm-miR156w --UUGACAGAAGAGA--GAGAGCAC-- 21

mdm-miR156v --UUGACAGAAGAGA--GAGAGCAC-- 21

mdm-miR156t --UUGACAGAAGAGA--GAGAGCAC-- 21

mdm-miR156u --UUGACAGAAGAGA--GAGAGCAC-- 21

gma-miR156t --UUGACAGAAGAAA--GGGAGCAC-- 21

gma-miR156p --UUGACAGAAGAAA--GGGAGCAC-- 21

ath-miR156h ---UGACAGAAGAAA--GAGAGCAC-- 20

mdm-miR156p --CUGACAGAAGAUA--GAGAGCAC-- 21

mdm-miR156q --CUGACAGAAGAUA--GAGAGCAC-- 21

mdm-miR156s --CUGACAGAAGAUA--GAGAGCAC-- 21

mdm-miR156r --CUGACAGAAGAUA--GAGAGCAC-- 21

hbr-miR156 --UUGACAGAAGAUA--GAGAGC---- 19

gma-miR156r --CUGACAGAAGAUA--GAGAGCAU-- 21

nta-miR156g ---UGACAGAAGAUA--GAGAGCAC-- 20

nta-miR156h ---UGACAGAAGAUA--GAGAGCAC-- 20

cme-miR156f ---UGACAGAAGAUA--GAGAGCAC-- 20

mdm-miR156aa ---UGACAGAAGAUA--GAGAGCAC-- 20

cme-miR156e --UUGACAGAAGAUA--GAGGGCAC-- 21

ptc-miR156l --UUGACAGAAGAUG--GAGAGCAC-- 21

mac-miR156 -GUUGACAGAAGAUA--GAGAGCAC-- 22

cme-miR156b --UUGACAGAAGAUA--GAGAGCAC-- 21

cpa-miR156e --UUGACAGAAGAUA--GAGAGCAC-- 21

cpa-miR156f --UUGACAGAAGAUA--GAGAGCAC-- 21

mdm-miR156ac --UUGACAGAAGAUA--GAGAGCAC-- 21

mdm-miR156ab --UUGACAGAAGAUA--GAGAGCAC-- 21

ptc-miR156h --UUGACAGAAGAUA--GAGAGCAC-- 21

ptc-miR156j --UUGACAGAAGAUA--GAGAGCAC-- 21

ptc-miR156i --UUGACAGAAGAUA--GAGAGCAC-- 21

ptc-miR156g --UUGACAGAAGAUA--GAGAGCAC-- 21

gma-miR156i --UUGACAGAAGAUA--GAGAGCAC-- 21

gma-miR156c --UUGACAGAAGAUA--GAGAGCAC-- 21

gma-miR156l --UUGACAGAAGAUA--GAGAGCAC-- 21

gma-miR156m --UUGACAGAAGAUA--GAGAGCAC-- 21

gma-miR156d --UUGACAGAAGAUA--GAGAGCAC-- 21

gma-miR156j --UUGACAGAAGAUA--GAGAGCAC-- 21

gma-miR156e --UUGACAGAAGAUA--GAGAGCAC-- 21

vvi-miR156i --UUGACAGAAGAUA--GAGAGCAC-- 21

vvi-miR156f --UUGACAGAAGAUA--GAGAGCAC-- 21

vvi-miR156g --UUGACAGAAGAUA--GAGAGCAC-- 21

mac-miR156d --UUGACAGAAGAUA--GAGAGCAC-- 21

nta-miR156j ---UGACAGAAGAUA--GAGAGCAC-- 20

nta-miR156i ---UGACAGAAGAUA--GAGAGCAC-- 20

mdm-miR156y ---UGACAGAAGAUA--GAGAGCAC-- 20

mdm-miR156z ---UGACAGAAGAUA--GAGAGCAC-- 20

mdm-miR156x ---UGACAGAAGAUA--GAGAGCAC-- 20

aqc-miR156a ---UGACAGAAGAUA--GAGAGCAC-- 20

aqc-miR156b ---UGACAGAAGAUA--GAGAGCAC-- 20

mac-miR156g ---UGACAGAAGAUA--GAGAGCAC-- 20

gma-miR156g -----ACAGAAGAUA--GAGAGCACAG 20

bcy-miR156 -UUUGACAGAAGAUA--GAGAGCAC-- 22

gma-miR156aa -----AUUGGAGUGAA-GGGAGCU--- 18

gma-miR156z -----AUUGGAGUGAA-GGGAGCU--- 18

gma-miR156ab -----AUUUAAGUGAU-GGGAGCUCCG 21

ath-miR156f-3p -GCUCACU-CUCUAUCCGUCACC---- 21

zma-miR156l-3p -GCUCACUGCUCUAUCUGUCACC---- 22

zma-miR156e-3p -GCUCACUGCUCUCUCUGUCAUC---- 22

zma-miR156i-3p -GCUCACUGCUCUAUCUGUCAUC---- 22

zma-miR156h-3p -GCUCACUGCUCUUUCUGUCAUC---- 22

osa-miR156b-3p -GCUCACU-CUCUAUCUGUCAGC---- 21

osa-miR156l-3p -GCUCACUUCUCUUUCUGUCAGC---- 22

osa-miR156h-3p -GCUCACUUCUCUUUCUGUCAGC---- 22

osa-miR156f-3p -GCUCACUUCUCUUUCUGUCAGC---- 22

zma-miR156f-3p -GCUCACUUCUCUUUCUGUCAGC---- 22

zma-miR156g-3p -GCUCACUUCUCUUUCUGUCAGC---- 22

zma-miR156d-3p -GCUCACUUCUCUUUCUGUCAGC---- 22

mac-miR156a-3p -GCUCACUUCUCUUCCUGUCAGC---- 22

mac-miR156h-3p -GCUCACUUCUCUUCCUGUCAGC---- 22

osa-miR156g-3p -GCUCACUUCUCUCUCUGUCAGC---- 22

osa-miR156c-3p -GCUCACUUCUCUCUCUGUCAGC---- 22

zma-miR156a-3p -GCUCACUUCUCUCUCUGUCAGU---- 22

osa-miR156j-3p -GCUCGCUCCUCUUUCUGUCAGC---- 22

zma-miR156k-3p -GCUCGCUUCUCUUUCUGUCAGC---- 22

ath-miR156b-3p UGCUCACCUCUCUUUCUGUCAGU---- 23

zma-miR156b-3p -GCUCACC-CUCUAUCUGUCAGU---- 21

ath-miR156a-3p -GCUCACUGCUCUUUCUGUCAGA---- 22

ath-miR156c-3p -GCUCACUGCUCUAUCUGUCAGA---- 22

zma-miR156j-3p UGCUCUCUGCUCUCACUGUCAUC---- 23

ath-miR156d-3p -GCUCACU-CUCUUUUUGUCAUAAC-- 23

2. miR159

vvi-miR159c UUUGGAUU-----GAAG-GGAGCU---CUA--- 21

mbg-miR159 UUUGGAUU-----GAAG-GGAGCU---CUA--- 21

gma-miR159a-3p UUUGGAUU-----GAAG-GGAGCU---CUA--- 21

gma-miR159e-3p UUUGGAUU-----GAAG-GGAGCU---CUA--- 21

hbr-miR159a UUUGGAUU-----GAAG-GGAGCU---CUA--- 21

ptc-miR159a UUUGGAUU-----GAAG-GGAGCU---CUA--- 21

ptc-miR159b UUUGGAUU-----GAAG-GGAGCU---CUA--- 21

csi-miR159 UUUGGAUU-----GAAG-GGAGCU---CUA--- 21

cpa-miR159a UUUGGAUU-----GAAG-GGAGCU---CUA--- 21

cme-miR159a UUUGGAUU-----GAAG-GGAGCU---CUA--- 21

nta-miR159 UUUGGAUU-----GAAG-GGAGCU---CUA--- 21

ath-miR159a UUUGGAUU-----GAAG-GGAGCU---CUA--- 21

ath-miR159b-3p UUUGGAUU-----GAAG-GGAGCU---CUU--- 21

ath-miR159c UUUGGAUU-----GAAG-GGAGCU---CCU--- 21

cme-miR159b AUUGGAUU-----GAAG-GGAGCU---CCU--- 21

osa-miR159e AUUGGAUU-----GAAG-GGAGCU---CCU--- 21

osa-miR159d AUUGGAUU-----GAAG-GGAGCU---CCG--- 21

osa-miR159c AUUGGAUU-----GAAG-GGAGCU---CCA--- 21

zma-miR159e-3p AUUGGUUU-----GAAG-GGAGCU---CCA--- 21

cpa-miR159b CUUGGAUU-----GAAG-GGAGCU---CC---- 20

ptc-miR159d CUUGGAUU-----GAAG-GGAGCU---CCU--- 21

zma-miR159c-3p CUUGGAUU-----GAAG-GGAGCU---CCU--- 21

mdm-miR159b CUUGGAUU-----GAAG-GGAGCU---CC---- 20

sbi-miR159b CUUGGAUU-----GAAG-GGAGCU---CCU--- 21

mdm-miR159a CUUGGAUU-----GAAG-GGAGCU---CC---- 20

zma-miR159d-3p CUUGGAUU-----GAAG-GGAGCU---CCU--- 21

gma-miR159b-3p AUUGGAGU-----GAAG-GGAGCU---CCA--- 21

gma-miR159f-3p AUUGGAGU-----GAAG-GGAGCU---CCA--- 21

ptc-miR159c AUUGGAGU-----GAAG-GGAGCU---CGA--- 21

gma-miR159c AUUGGAGU-----GAAG-GGAGCU---CCG--- 21

vvi-miR159a CUUGGAGU-----GAAG-GGAGCU---CUC--- 21

vvi-miR159b CUUGGAGU-----GAAG-GGAGCU---CUC--- 21

ptc-miR159e CUUGGGGU-----GAAG-GGAGCU---C-CU-- 21

osa-miR159f CUUGGAUU-----GAAG-GGAGCU---CUA--- 21

osa-miR159a.1 UUUGGAUU-----GAAG-GGAGCU---CUG--- 21

osa-miR159b UUUGGAUU-----GAAG-GGAGCU---CUG--- 21

sbi-miR159a UUUGGAUU-----GAAG-GGAGCU---CUG--- 21

tae-miR159a UUUGGAUU-----GAAG-GGAGCU---CUG--- 21

tae-miR159b UUUGGAUU-----GAAG-GGAGCU---CUG--- 21

zma-miR159j-3p UUUGGAUU-----GAAG-GGAGCU---CUG--- 21

zma-miR159f-3p UUUGGAUU-----GAAG-GGAGCU---CUG--- 21

zma-miR159k-3p UUUGGAUU-----GAAG-GGAGCU---CUG--- 21

zma-miR159a-3p UUUGGAUU-----GAAG-GGAGCU---CUG--- 21

zma-miR159b-3p UUUGGAUU-----GAAG-GGAGCU---CUG--- 21

aqc-miR159 UUUGGACU-----GAAG-GGAGCU---CUA--- 21

zma-miR159i-3p UUUGGAGU-----GAAG-GGAGCU---CUG--- 21

zma-miR159h-3p UUUGGAGU-----GAAG-GGAGCU---CUG--- 21

zma-miR159g-3p UUUGGAGU-----GAAG-GGAGUU---CUG--- 21

ath-miR159b-5p ----GAG--CU---CCU-UG--AAGUUCAAUGG 21

gma-miR159a-5p ----GAG--CU---CCU-UG--AAGUCCAAUUG 21

gma-miR159e-5p ----GAG--CU---CCU-UG--AAGUCCAAUU- 20

zma-miR159k-5p ----GUG--CU---CC---CUUCAAACCAAUAA 21

zma-miR159b-5p ----GUG--CU---CC---CUUCAAACCAAUAA 21

zma-miR159j-5p ----GUG--CU---CC---CUUCAAACCAAUAA 21

zma-miR159g-5p ----GUG--CU---CC---CUUCACACCAAUAA 21

zma-miR159h-5p ----GUG--CU---CC---CUUCACACCAAUAA 21

zma-miR159i-5p ----GUG--CU---CC---CUUCACACCAAUAA 21

zma-miR159d-5p ----GAG--CU---CC---CUUCGAUCCAAUCC 21

zma-miR159c-5p ----GAG--CU---CC---CUUCGAUCCAAUCC 21

zma-miR159a-5p ----GAG--CU---CCU--AU-CAUUCCAAUGA 21

zma-miR159f-5p ----GAG--CU---CCU--CU-CAUUCCAAUGA 21

gma-miR159b-5p ----GAGUUC----CCUG----CACUCCAAGUC 21

gma-miR159f-5p ----GAGUUC----CCUG----CACUCCAAGUC 21

mdm-miR159c ----GAAU--U---CCU-UCUCCUCUCCUUU-- 21

hbr-miR159b --UUGCAUAU----CUCAGGAGCUU--CA---- 21

osa-miR159a.2 --UUGCAUGC----CCCAGGAGCUG--CA---- 21

gma-miR159d ---AGCU-GCUUAGCUAUGGAUC---CC----- 21

zma-miR159e-5p --CAGCU-CCU--GC-A-GCAUCUGUUC----- 21

* . *

3. miR160

mac-miR160a UGCCU-----GGCUCCCUGUAU-GCCA-- 21

mac-miR160g-5p UGCCU-----GGCUCCCUGUAU-GCCA-- 21

zma-miR160b-5p UGCCU-----GGCUCCCUGUAU-GCCA-- 21

zma-miR160e UGCCU-----GGCUCCCUGUAU-GCCA-- 21

zma-miR160c-5p UGCCU-----GGCUCCCUGUAU-GCCA-- 21

zma-miR160d-5p UGCCU-----GGCUCCCUGUAU-GCCA-- 21

zma-miR160g-5p UGCCU-----GGCUCCCUGUAU-GCCA-- 21

zma-miR160a-5p UGCCU-----GGCUCCCUGUAU-GCCA-- 21

sbi-miR160b UGCCU-----GGCUCCCUGUAU-GCCA-- 21

tae-miR160 UGCCU-----GGCUCCCUGUAU-GCCA-- 21

sbi-miR160a UGCCU-----GGCUCCCUGUAU-GCCA-- 21

sbi-miR160e UGCCU-----GGCUCCCUGUAU-GCCA-- 21

sbi-miR160c UGCCU-----GGCUCCCUGUAU-GCCA-- 21

osa-miR160d-5p UGCCU-----GGCUCCCUGUAU-GCCA-- 21

osa-miR160a-5p UGCCU-----GGCUCCCUGUAU-GCCA-- 21

sbi-miR160d UGCCU-----GGCUCCCUGUAU-GCCA-- 21

osa-miR160c-5p UGCCU-----GGCUCCCUGUAU-GCCA-- 21

osa-miR160b-5p UGCCU-----GGCUCCCUGUAU-GCCA-- 21

vvi-miR160c UGCCU-----GGCUCCCUGUAU-GCCA-- 21

vvi-miR160e UGCCU-----GGCUCCCUGUAU-GCCA-- 21

vvi-miR160d UGCCU-----GGCUCCCUGUAU-GCCA-- 21

gma-miR160a-5p UGCCU-----GGCUCCCUGUAU-GCCA-- 21

gma-miR160f UGCCU-----GGCUCCCUGUAU-GCCA-- 21

aqc-miR160b UGCCU-----GGCUCCCUGUAU-GCCA-- 21

ptc-miR160d UGCCU-----GGCUCCCUGUAU-GCCA-- 21

ptc-miR160b-5p UGCCU-----GGCUCCCUGUAU-GCCA-- 21

ptc-miR160c-5p UGCCU-----GGCUCCCUGUAU-GCCA-- 21

ptc-miR160a UGCCU-----GGCUCCCUGUAU-GCCA-- 21

csi-miR160 -GCCU-----GGCUCCCUGUAU-GCCAU- 21

mdm-miR160b UGCCU-----GGCUCCCUGUAU-GCCA-- 21

mdm-miR160d UGCCU-----GGCUCCCUGUAU-GCCA-- 21

mdm-miR160c UGCCU-----GGCUCCCUGUAU-GCCA-- 21

mdm-miR160a UGCCU-----GGCUCCCUGUAU-GCCA-- 21

mdm-miR160e UGCCU-----GGCUCCCUGUAU-GCCA-- 21

cpa-miR160c-5p UGCCU-----GGCUCCCUGUAU-GCCA-- 21

cpa-miR160e UGCCU-----GGCUCCCUGUAU-GCCA-- 21

cpa-miR160f-5p UGCCU-----GGCUCCCUGUAU-GCCA-- 21

cpa-miR160b UGCCU-----GGCUCCCUGUAU-GCCA-- 21

cpa-miR160a UGCCU-----GGCUCCCUGUAU-GCCA-- 21

cme-miR160c UGCCU-----GGCUCCCUGUAU-GCCA-- 21

cme-miR160b UGCCU-----GGCUCCCUGUAU-GCCA-- 21

cme-miR160a UGCCU-----GGCUCCCUGUAU-GCCA-- 21

nta-miR160c UGCCU-----GGCUCCCUGUAU-GCCA-- 21

nta-miR160a UGCCU-----GGCUCCCUGUAU-GCCA-- 21

nta-miR160b UGCCU-----GGCUCCCUGUAU-GCCA-- 21

ath-miR160c-5p UGCCU-----GGCUCCCUGUAU-GCCA-- 21

ath-miR160b UGCCU-----GGCUCCCUGUAU-GCCA-- 21

ath-miR160a-5p UGCCU-----GGCUCCCUGUAU-GCCA-- 21

osa-miR160e-5p UGCCU-----GGCUCCCUGUAU-GCCG-- 21

zma-miR160f-5p UGCCU-----GGCUCCCUGUAU-GCCG-- 21

gma-miR160b UGCCU-----GGCUCCCUGUAU-GCC--- 20

gma-miR160c UGCCU-----GGCUCCCUGUAU-GCC--- 20

gma-miR160e UGCCU-----GGCUCCCUGUAU-GCC--- 20

gma-miR160d UGCCU-----GGCUCCCUGUAU-GCC--- 20

cme-miR160d UGCCU-----GGCUCCCUGAAU-GCCA-- 21

cpa-miR160d UGCCU-----GGCUCCCUGAAU-GCCA-- 21

ptc-miR160f UGCCU-----GGCUCCCUGAAU-GCCA-- 21

ptc-miR160e-5p UGCCU-----GGCUCCCUGAAU-GCCA-- 21

vvi-miR160a UGCCU-----GGCUCCCUGAAU-GCCAUC 23

vvi-miR160b UGCCU-----GGCUCCCUGAAU-GCCAUC 23

osa-miR160f-5p UGCCU-----GGCUCCCUGAAU-GCCA-- 21

sbi-miR160f UGCCU-----GGCUCCCUGAAU-GCCA-- 21

ptc-miR160g UGCCU-----GGCUCCCUGGAU-GCCA-- 21

aqc-miR160a UGCCU-----GGCUCCCUGGAU-GCCA-- 21

ptc-miR160h UGCCU-----GGCUCCCUGCAU-GCCA-- 21

nta-miR160d UGCCU-----GGCUCCCUGCAU-GCCA-- 21

ath-miR160a-3p -GCG-UAUGAGGAGCCAUGCAU-A----- 21

cpa-miR160c-3p -GCG-UAUGAGGAGCCAUGCAU-A----- 21

cpa-miR160f-3p -GCG-UAUGAGGAGCCAUGCAU-A----- 21

ptc-miR160b-3p -GCG-UAUGAGGAGCCAUGCAU-A----- 21

ptc-miR160c-3p -GCG-UAUGAGGAGCCAUGCAU-A----- 21

gma-miR160a-3p -GCG-UAUGAGGAGCCAAGCAU-A----- 21

osa-miR160c-3p -GCG-UGCACGGAGCCAAGCAU-A----- 21

osa-miR160d-3p -GCG-UGCGAGGAGCCAAGCAU-G----- 21

osa-miR160b-3p -GCG-UGCAAGGAGCCAAGCAU-G----- 21

osa-miR160a-3p -GCG-UGCAAGGAGCCAAGCAU-G----- 21

zma-miR160b-3p -GCG-UGCAAGGAGCCAAGCAU-G----- 21

zma-miR160g-3p -GCG-UGCAAGGAGCCAAGCAU-G----- 21

mac-miR160g* -GCG-UGCAAGGAGCCAAGCAU-G----- 21

zma-miR160a-3p -GCG-UGCAAGGGGCCAAGCAU-G----- 21

ath-miR160c-3p --CG-UACAAGGAGUCAAGCAUGA----- 21

ptc-miR160e-3p -GCAU-GAGGGGAGUCGAGCAG-G----- 21

osa-miR160f-3p -GCAUUGAGGG-AGUCAUGCAG-G----- 21

osa-miR160e-3p -GCG-UGCGAGGUGCCAAGCAU-G----- 21

zma-miR160f-3p -GCG-UGCGAGGUGCCAGGCAU-G----- 21

zma-miR160c-3p -GCG-UGCAUGGUGCCAAGCAU-A----- 21

zma-miR160d-3p -GCG-UGCGUGGAGCCAAGCAU-G----- 21

ghr-miR160 -----UAUGAGGAGCCAUGCAU-GUAU-- 21

* * * * .

4. miR162

osa-miR162a UCGAUAAACCUCUGCAUCCAG- 21

sbi-miR162 UCGAUAAACCUCUGCAUCCAG- 21

vvi-miR162 UCGAUAAACCUCUGCAUCCAG- 21

gma-miR162c UCGAUAAACCUCUGCAUCCAG- 21

gma-miR162b UCGAUAAACCUCUGCAUCCAG- 21

ptc-miR162b UCGAUAAACCUCUGCAUCCAG- 21

ptc-miR162a UCGAUAAACCUCUGCAUCCAG- 21

csi-miR162-3p UCGAUAAACCUCUGCAUCCAG- 21

mdm-miR162b UCGAUAAACCUCUGCAUCCAG- 21

mdm-miR162a UCGAUAAACCUCUGCAUCCAG- 21

cpa-miR162a UCGAUAAACCUCUGCAUCCAG- 21

cme-miR162 UCGAUAAACCUCUGCAUCCAG- 21

nta-miR162b UCGAUAAACCUCUGCAUCCAG- 21

nta-miR162a UCGAUAAACCUCUGCAUCCAG- 21

ghr-miR162a UCGAUAAACCUCUGCAUCCAG- 21

ath-miR162b-3p UCGAUAAACCUCUGCAUCCAG- 21

ath-miR162a-3p UCGAUAAACCUCUGCAUCCAG- 21

zma-miR162-3p UCGAUAAACCUCUGCAUCCA-- 20

gma-miR162a UCGAUAAACCUCUGCAUCCA-- 20

osa-miR162b UCGAUAAGCCUCUGCAUCCAG- 21

mac-miR162b UCGAUAAACCGCUGCGUCCAG- 21

mac-miR162 UCGAUAAACCGCUGCGUCCA-- 20

ath-miR162b-5p UGGAGGCAGCGGUUCAUCGAUC 22

ath-miR162a-5p UGGAGGCAGCGGUUCAUCGAUC 22

csi-miR162-5p UGGAGGCAGCGGUUCAUCGAUC 22

zma-miR162-5p -GGGCGCAGUGGUUUAUCGAUC 21

mac-miR162* -GGAUGCAGAGGUUUAUCGACC 21

* * ** *

5. miR164

zma-miR164c-5p UGGAG---AAGCAGGG-CACGUGCA 21

mac-miR164e UGGAG---AAGCAGGG-CACGUGCA 21

zma-miR164d-5p UGGAG---AAGCAGGG-CACGUGCA 21

zma-miR164g-5p UGGAG---AAGCAGGG-CACGUGCA 21

sbi-miR164a UGGAG---AAGCAGGG-CACGUGCA 21

tae-miR164 UGGAG---AAGCAGGG-CACGUGCA 21

zma-miR164b-5p UGGAG---AAGCAGGG-CACGUGCA 21

zma-miR164a-5p UGGAG---AAGCAGGG-CACGUGCA 21

sbi-miR164e UGGAG---AAGCAGGG-CACGUGCA 21

osa-miR164f UGGAG---AAGCAGGG-CACGUGCA 21

sbi-miR164d UGGAG---AAGCAGGG-CACGUGCA 21

osa-miR164a UGGAG---AAGCAGGG-CACGUGCA 21

osa-miR164b UGGAG---AAGCAGGG-CACGUGCA 21

vvi-miR164c UGGAG---AAGCAGGG-CACGUGCA 21

vvi-miR164a UGGAG---AAGCAGGG-CACGUGCA 21

vvi-miR164d UGGAG---AAGCAGGG-CACGUGCA 21

gma-miR164j UGGAG---AAGCAGGG-CACGUGCA 21

gma-miR164a UGGAG---AAGCAGGG-CACGUGCA 21

gma-miR164f UGGAG---AAGCAGGG-CACGUGCA 21

gma-miR164k UGGAG---AAGCAGGG-CACGUGCA 21

gma-miR164e UGGAG---AAGCAGGG-CACGUGCA 21

gma-miR164h UGGAG---AAGCAGGG-CACGUGCA 21

ptc-miR164a UGGAG---AAGCAGGG-CACGUGCA 21

gma-miR164i UGGAG---AAGCAGGG-CACGUGCA 21

gma-miR164g UGGAG---AAGCAGGG-CACGUGCA 21

ptc-miR164e UGGAG---AAGCAGGG-CACGUGCA 21

ptc-miR164d UGGAG---AAGCAGGG-CACGUGCA 21

ptc-miR164c UGGAG---AAGCAGGG-CACGUGCA 21

ptc-miR164b UGGAG---AAGCAGGG-CACGUGCA 21

ssl-miR164b UGGAG---AAGCAGGG-CACGUGCA 21

ssl-miR164a UGGAG---AAGCAGGG-CACGUGCA 21

mdm-miR164e UGGAG---AAGCAGGG-CACGUGCA 21

mdm-miR164c UGGAG---AAGCAGGG-CACGUGCA 21

mdm-miR164f UGGAG---AAGCAGGG-CACGUGCA 21

mdm-miR164b UGGAG---AAGCAGGG-CACGUGCA 21

mdm-miR164d UGGAG---AAGCAGGG-CACGUGCA 21

csi-miR164 UGGAG---AAGCAGGG-CACGUGCA 21

cpa-miR164c UGGAG---AAGCAGGG-CACGUGCA 21

cpa-miR164b UGGAG---AAGCAGGG-CACGUGCA 21

cpa-miR164a UGGAG---AAGCAGGG-CACGUGCA 21

cme-miR164d UGGAG---AAGCAGGG-CACGUGCA 21

cme-miR164c UGGAG---AAGCAGGG-CACGUGCA 21

nta-miR164a UGGAG---AAGCAGGG-CACGUGCA 21

nta-miR164b UGGAG---AAGCAGGG-CACGUGCA 21

ghr-miR164 UGGAG---AAGCAGGG-CACGUGCA 21

ath-miR164a UGGAG---AAGCAGGG-CACGUGCA 21

ath-miR164b-5p UGGAG---AAGCAGGG-CACGUGCA 21

osa-miR164c UGGAG---AAGCAGGG-UACGUGCA 21

sbi-miR164c UGGAG---AAGCAGGA-CACGUGAG 21

zma-miR164e-5p UGGAG---AAGCAGGA-CACGUGAG 21

osa-miR164e UGGAG---AAGCAGGG-CACGUGAG 21

zma-miR164h-5p UGGAG---AAGCAGGG-CACGUGUG 21

ath-miR164c-5p UGGAG---AAGCAGGG-CACGUGCG 21

cme-miR164a UGGAG---AAGCAGGG-CACGUGCU 21

gma-miR164c UGGAG---AAGCAGGG-CACGUGC- 20

zma-miR164f-5p UGGAG---AAGCAGGG-CACGUGCU 21

gma-miR164d UGGAG---AAGCAGGG-CACGUGC- 20

osa-miR164d UGGAG---AAGCAGGG-CACGUGCU 21

gma-miR164b UGGAG---AAGCAGGG-CACGUGC- 20

sbi-miR164b UGGAG---AAGCAGGG-CACGUGCU 21

nta-miR164c UGGAG---AAGCAGGG-CACAUGCU 21

ptc-miR164f UGGAG---AAGCAGGG-CACAUGCU 21

vvi-miR164b UGGAG---AAGCAGGG-CACAUGCU 21

mdm-miR164a UGGAG---AAGCAGGG-CACAUGCC 21

cme-miR164b UGGAG---AGGCAGGG-CACAUGCU 21

cpa-miR164d UGGAG---AAGGGGAG-CACGUGCA 21

cpa-miR164e UGGAG---AAGGGGAG-CACGUGCA 21

zma-miR164c-3p -CAUGUG-CC-CUUCUUCUCCAUC- 21

zma-miR164h-3p -CAUGUG-CC-CUUCUUCUCCAUC- 21

ath-miR164b-3p -CAUGUG-CC-CAUCUUCACCAUC- 21

zma-miR164b-3p --AUGUG-CC-CAUCUUCUCCACC- 20

zma-miR164e-3p -CAUGUGUCCGCC-CU-CUCCACC- 21

zma-miR164a-3p -CACGUGUUCUCCUU--CUCCAUC- 21

zma-miR164d-3p -CACGUGGUCUCCUU--CUCCAU-- 20

zma-miR164g-3p -CACGUGCUCCCCUU--CUCCACC- 21

zma-miR164f-3p -CACGUGCGCUCCUU--CUCCAAC- 21

ath-miR164c-3p -CACGUGUUCUACUA--CUCCAAC- 21

. * *

6. miR166

cpa-miR166e ----GGACCAG--GCUUCAUUCCCC--- 19

gma-miR166m ---CGGACCAG--GCUUCAUUCCCC--- 20

csi-miR166a --UCGGACCAG--GCUUCAUUCCCCC-- 22

mac-miR166 --UCGGACCAG--GCUUCAUUCCCCC-- 22

ath-miR166d --UCGGACCAG--GCUUCAUUCCCC--- 21

osa-miR166e-3p --UCGAACCAG--GCUUCAUUCCCC--- 21

ath-miR166b-3p --UCGGACCAG--GCUUCAUUCCCC--- 21

ath-miR166e-3p --UCGGACCAG--GCUUCAUUCCCC--- 21

ath-miR166c --UCGGACCAG--GCUUCAUUCCCC--- 21

ath-miR166f --UCGGACCAG--GCUUCAUUCCCC--- 21

ath-miR166a-3p --UCGGACCAG--GCUUCAUUCCCC--- 21

ath-miR166g --UCGGACCAG--GCUUCAUUCCCC--- 21

ghr-miR166b --UCGGACCAG--GCUUCAUUCCCC--- 21

nta-miR166c --UCGGACCAG--GCUUCAUUCCCC--- 21

nta-miR166f --UCGGACCAG--GCUUCAUUCCCC--- 21

nta-miR166b --UCGGACCAG--GCUUCAUUCCCC--- 21

nta-miR166d --UCGGACCAG--GCUUCAUUCCCC--- 21

nta-miR166h --UCGGACCAG--GCUUCAUUCCCC--- 21

nta-miR166g --UCGGACCAG--GCUUCAUUCCCC--- 21

cme-miR166h --UCGGACCAG--GCUUCAUUCCCC--- 21

cme-miR166a --UCGGACCAG--GCUUCAUUCCCC--- 21

cpa-miR166c --UCGGACCAG--GCUUCAUUCCCC--- 21

aqc-miR166e --UCGGACCAG--GCUUCAUUCCCC--- 21

gma-miR166d --UCGGACCAG--GCUUCAUUCCCC--- 21

vvi-miR166h --UCGGACCAG--GCUUCAUUCCCC--- 21

osa-miR166b-3p --UCGGACCAG--GCUUCAUUCCCC--- 21

osa-miR166d-3p --UCGGACCAG--GCUUCAUUCCCC--- 21

osa-miR166j-3p --UCGGACCAG--GCUUCAUUCCCC--- 21

osa-miR166a-3p --UCGGACCAG--GCUUCAUUCCCC--- 21

osa-miR166f --UCGGACCAG--GCUUCAUUCCCC--- 21

zma-miR166a-3p --UCGGACCAG--GCUUCAUUCCCC--- 21

mac-miR166c-3p --UCGGACCAG--GCUUCAUUCCCC--- 21

osa-miR166c-3p --UCGGACCAG--GCUUCAUUCCCC--- 21

vvi-miR166e --UCGGACCAG--GCUUCAUUCCCC--- 21

vvi-miR166f --UCGGACCAG--GCUUCAUUCCCC--- 21

vvi-miR166d --UCGGACCAG--GCUUCAUUCCCC--- 21

vvi-miR166g --UCGGACCAG--GCUUCAUUCCCC--- 21

vvi-miR166c --UCGGACCAG--GCUUCAUUCCCC--- 21

gma-miR166e --UCGGACCAG--GCUUCAUUCCCC--- 21

gma-miR166f --UCGGACCAG--GCUUCAUUCCCC--- 21

gma-miR166o --UCGGACCAG--GCUUCAUUCCCC--- 21

gma-miR166g --UCGGACCAG--GCUUCAUUCCCC--- 21

gma-miR166a-3p --UCGGACCAG--GCUUCAUUCCCC--- 21

gma-miR166i-3p --UCGGACCAG--GCUUCAUUCCCC--- 21

gma-miR166c-3p --UCGGACCAG--GCUUCAUUCCCC--- 21

gma-miR166b --UCGGACCAG--GCUUCAUUCCCC--- 21

gma-miR166n --UCGGACCAG--GCUUCAUUCCCC--- 21

aqc-miR166b --UCGGACCAG--GCUUCAUUCCCC--- 21

ptc-miR166l --UCGGACCAG--GCUUCAUUCCCC--- 21

ptc-miR166g --UCGGACCAG--GCUUCAUUCCCC--- 21

ptc-miR166i --UCGGACCAG--GCUUCAUUCCCC--- 21

ptc-miR166h --UCGGACCAG--GCUUCAUUCCCC--- 21

ptc-miR166f --UCGGACCAG--GCUUCAUUCCCC--- 21

ptc-miR166d --UCGGACCAG--GCUUCAUUCCCC--- 21

ptc-miR166m --UCGGACCAG--GCUUCAUUCCCC--- 21

ptc-miR166c --UCGGACCAG--GCUUCAUUCCCC--- 21

ptc-miR166b --UCGGACCAG--GCUUCAUUCCCC--- 21

ptc-miR166e --UCGGACCAG--GCUUCAUUCCCC--- 21

ptc-miR166a --UCGGACCAG--GCUUCAUUCCCC--- 21

ptc-miR166k --UCGGACCAG--GCUUCAUUCCCC--- 21

ptc-miR166j --UCGGACCAG--GCUUCAUUCCCC--- 21

ssl-miR166b --UCGGACCAG--GCUUCAUUCCCC--- 21

csi-miR166e-3p --UCGGACCAG--GCUUCAUUCCCC--- 21

mdm-miR166a --UCGGACCAG--GCUUCAUUCCCC--- 21

mdm-miR166f --UCGGACCAG--GCUUCAUUCCCC--- 21

mdm-miR166d --UCGGACCAG--GCUUCAUUCCCC--- 21

mdm-miR166e --UCGGACCAG--GCUUCAUUCCCC--- 21

mdm-miR166c --UCGGACCAG--GCUUCAUUCCCC--- 21

mdm-miR166g --UCGGACCAG--GCUUCAUUCCCC--- 21

mdm-miR166b --UCGGACCAG--GCUUCAUUCCCC--- 21

mdm-miR166h --UCGGACCAG--GCUUCAUUCCCC--- 21

mdm-miR166i --UCGGACCAG--GCUUCAUUCCCC--- 21

cpa-miR166b --UCGGACCAG--GCUUCAUUCCCC--- 21

cpa-miR166a --UCGGACCAG--GCUUCAUUCCCC--- 21

cme-miR166d --UCGGACCAG--GCUUCAUUCCCC--- 21

cme-miR166f --UCGGACCAG--GCUUCAUUCCCC--- 21

cme-miR166b --UCGGACCAG--GCUUCAUUCCCC--- 21

cme-miR166c --UCGGACCAG--GCUUCAUUCCCC--- 21

nta-miR166e --UCGGACCAG--GCUUCAUUCCCC--- 21

nta-miR166a --UCGGACCAG--GCUUCAUUCCCC--- 21

zma-miR166g-3p --UCGGACCAG--GCUUCAUUCCC---- 20

zma-miR166e --UCGGACCAG--GCUUCAUUCCC---- 20

zma-miR166b-3p --UCGGACCAG--GCUUCAUUCCC---- 20

zma-miR166d-3p --UCGGACCAG--GCUUCAUUCCC---- 20

zma-miR166h-3p --UCGGACCAG--GCUUCAUUCCC---- 20

zma-miR166c-3p --UCGGACCAG--GCUUCAUUCCC---- 20

zma-miR166i-3p --UCGGACCAG--GCUUCAUUCCC---- 20

zma-miR166f --UCGGACCAG--GCUUCAUUCCC---- 20

sbi-miR166j --UCGGACCAG--GCUUCAUUCCC---- 20

sbi-miR166h --UCGGACCAG--GCUUCAUUCCC---- 20

sbi-miR166a --UCGGACCAG--GCUUCAUUCCC---- 20

sbi-miR166b --UCGGACCAG--GCUUCAUUCCC---- 20

sbi-miR166c --UCGGACCAG--GCUUCAUUCCC---- 20

sbi-miR166d --UCGGACCAG--GCUUCAUUCCC---- 20

sbi-miR166i --UCGGACCAG--GCUUCAUUCCC---- 20

gma-miR166q --UCGGACCAG--GCUUCAUUCCC---- 20

gma-miR166t --UCGGACCAG--GCUUCAUUCCC---- 20

gma-miR166s --UCGGACCAG--GCUUCAUUCCC---- 20

gma-miR166p --UCGGACCAG--GCUUCAUUCCC---- 20

gma-miR166r --UCGGACCAG--GCUUCAUUCCC---- 20

cme-miR166e --UCGGACCAG--GCUUCAUUCCUC--- 21

osa-miR166i-3p --UCGGAUCAG--GCUUCAUUCCUC--- 21

ssl-miR166a --UCGGACCAG--GCUUCAUUCCUC--- 21

aqc-miR166a --UCGGACCAG--GCUUCAUUCCUC--- 21

aqc-miR166d --UCGGACCAG--GCUUCAUUCCUC--- 21

osa-miR166g-3p --UCGGACCAG--GCUUCAUUCCUC--- 21

osa-miR166h-3p --UCGGACCAG--GCUUCAUUCCUC--- 21

sbi-miR166f --UCGGACCAG--GCUUCAUUCCUC--- 21

zma-miR166l-3p --UCGGACCAG--GCUUCAUUCCUC--- 21

zma-miR166m-3p --UCGGACCAG--GCUUCAUUCCUC--- 21

ptc-miR166q --UCGGACCAG--GCUUCAUUCCUU--- 21

ptc-miR166p --UCGGACCAG--GCUCCAUUCCUU--- 21

ptc-miR166o --UCGGACCAG--GCUUCAUUCCUU--- 21

ptc-miR166n --UCGGACCAG--GCUUCAUUCCUU--- 21

aqc-miR166c --UCGGACCAG--GCUUCAUUCCU---- 20

sbi-miR166k --UCGGACCAG--GCUUCAUUCCU---- 20

gma-miR166h-3p UCUCGGACCAG--GCUUCAUUCC----- 21

gma-miR166u UCUCGGACCAG--GCUUCAUUC------ 20

gma-miR166k UCUCGGACCAG--GCUUCAUUCC----- 21

vvi-miR166b --UCGGACCAG--GCUUCAUUCC----- 19

vvi-miR166a --UCGGACCAG--GCUUCAUUCC----- 19

csi-miR166c --UCGGACCAG--GCUUCAUUCCC---- 20

cpa-miR166d --UCGGACCAG--GCUUCAUUCCCG--- 21

csi-miR166b --UCGGACCAG--GCUUCAUUCCCGU-- 22

gma-miR166j-3p --UCGGACCAG--GCUUCAUUCCCG--- 21

cme-miR166g --UCGGACCAG--GCUUCAUUCCC---- 20

csi-miR166d --UCGGACCAG--GCUUCAUUCCCU--- 21

osa-miR166m --UCGGACCAG--GCUUCAUUCCCU--- 21

cme-miR166i --UCGGACCAG--GCUUCAUUCUC---- 20

osa-miR166l-3p --UCGGACCAG--GCUUCAAUCCCU--- 21

osa-miR166k-3p --UCGGACCAG--GCUUCAAUCCCU--- 21

sbi-miR166e --UCGGACCAG--GCUUCAAUCCCU--- 21

sbi-miR166g --UCGGACCAG--GCUUCAAUCCCU--- 21

zma-miR166j-3p --UCGGACCAG--GCUUCAAUCCCU--- 21

zma-miR166k-3p --UCGGACCAG--GCUUCAAUCCCU--- 21

zma-miR166n-3p --UCGGACCAG--GCUUCAAUCCCU--- 21

ath-miR166b-5p ----GGACUGUU-GUCUGGCUCGAGG-- 21

ath-miR166a-5p ----GGACUGUU-GUCUGGCUCGAGG-- 21

gma-miR166j-5p ----GGAAUGUU-GUUUGGCUCGAGG-- 21

gma-miR166h-5p ----GGAAUGUU-GUUUGGCUCGAGG-- 21

zma-miR166g-5p ----GGAAUGUU-GUCUGGUUGGAGA-- 21

osa-miR166k-5p ----GGUUUGUU-GUCUGGCUCGAGG-- 21

zma-miR166j-5p ----GGUUUGUUUGUCUGGUUCAAGG-- 22

csi-miR166e-5p ----GGAAUGUU-GUCUGGCUCGAGG-- 21

gma-miR166l ----GGAAUGUU-GUCUGGCUCGAGG-- 21

gma-miR166c-5p ----GGAAUGUU-GUCUGGCUCGAGG-- 21

gma-miR166a-5p ----GGAAUGUU-GUCUGGCUCGAGG-- 21

osa-miR166d-5p ----GGAAUGUU-GUCUGGCUCGAGG-- 21

zma-miR166c-5p ----GGAAUGUU-GUCUGGCUCGAGG-- 21

mac-miR166c-5p ----GGAAUGUU-GUCUGGCUCGAGG-- 21

ath-miR166e-5p ----GGAAUGUU-GUCUGGCACGAGG-- 21

osa-miR166b-5p ----GGAAUGUU-GUCUGGCUCGGGG-- 21

zma-miR166a-5p ----GGAAUGUU-GUCUGGCUCGGGG-- 21

mac-miR166b ----GGAAUGUU-GUCUGGCUCGGGG-- 21

osa-miR166h-5p ----GGAAUGUU-GGCUGGCUCGAGG-- 21

zma-miR166m-5p ----GGAAUGUU-GGCUGGCUCGAGG-- 21

zma-miR166k-5p ----GGAUUGUU-GUCUGGCUCGGGG-- 21

zma-miR166n-5p ----GGAUUGUU-GUCUGGCUCGGUG-- 21

osa-miR166c-5p ----GGAAUGUU-GUCUGGUCCGAG--- 20

zma-miR166i-5p ----GGAAUGUC-GUCUGGCGCGAGA-- 21

gma-miR166i-5p ----GGAAUGUC-GUCUGGUUCGAG--- 20

osa-miR166a-5p ----GGAAUGUU-GUCUGGUUCAAGG-- 21

osa-miR166e-5p ----GGAAUGUU-GUCUGGUUCAAGG-- 21

zma-miR166b-5p ----GGAAUGUU-GUCUGGUUCAAGG-- 21

zma-miR166d-5p ----GGAAUGUU-GUCUGGUUCAAGG-- 21

osa-miR166l-5p ----GGAUUGUU-GUCUGGUUCAAGG-- 21

osa-miR166g-5p ------AAUGGA-GGCUGAUCCAAGAUC 21

zma-miR166l-5p -----GAAUGGA-GGCUGGUCCAAGA-- 20

osa-miR166i-5p ------AAUGCA-GUUUGAUCCAAGAUC 21

osa-miR166j-5p -----GAAUGAC-GUCCGGUCUGAAGA- 21

zma-miR166h-5p ----GGAAUGAC-GUCCGGUCCGAAC-- 21

*

7. miR167

sbi-miR167b ----UGAAGC----UGCCAGCAUGAUCUA-- 21

tae-miR167a ----UGAAGC----UGCCAGCAUGAUCUA-- 21

sbi-miR167i ----UGAAGC----UGCCAGCAUGAUCUA-- 21

sbi-miR167a ----UGAAGC----UGCCAGCAUGAUCUA-- 21

osa-miR167b ----UGAAGC----UGCCAGCAUGAUCUA-- 21

osa-miR167c-5p ----UGAAGC----UGCCAGCAUGAUCUA-- 21

osa-miR167a-5p ----UGAAGC----UGCCAGCAUGAUCUA-- 21

vvi-miR167b ----UGAAGC----UGCCAGCAUGAUCUA-- 21

vvi-miR167e ----UGAAGC----UGCCAGCAUGAUCUA-- 21

vvi-miR167d ----UGAAGC----UGCCAGCAUGAUCUA-- 21

gma-miR167a ----UGAAGC----UGCCAGCAUGAUCUA-- 21

gma-miR167b ----UGAAGC----UGCCAGCAUGAUCUA-- 21

gma-miR167d ----UGAAGC----UGCCAGCAUGAUCUA-- 21

ptc-miR167d ----UGAAGC----UGCCAGCAUGAUCUA-- 21

ptc-miR167b ----UGAAGC----UGCCAGCAUGAUCUA-- 21

ptc-miR167a ----UGAAGC----UGCCAGCAUGAUCUA-- 21

ptc-miR167c ----UGAAGC----UGCCAGCAUGAUCUA-- 21

mdm-miR167b ----UGAAGC----UGCCAGCAUGAUCUA-- 21

mdm-miR167f ----UGAAGC----UGCCAGCAUGAUCUA-- 21

mdm-miR167g ----UGAAGC----UGCCAGCAUGAUCUA-- 21

mdm-miR167d ----UGAAGC----UGCCAGCAUGAUCUA-- 21

mdm-miR167c ----UGAAGC----UGCCAGCAUGAUCUA-- 21

mdm-miR167e ----UGAAGC----UGCCAGCAUGAUCUA-- 21

cpa-miR167b ----UGAAGC----UGCCAGCAUGAUCUA-- 21

cpa-miR167a ----UGAAGC----UGCCAGCAUGAUCUA-- 21

cme-miR167b ----UGAAGC----UGCCAGCAUGAUCUA-- 21

cme-miR167a ----UGAAGC----UGCCAGCAUGAUCUA-- 21

nta-miR167e ----UGAAGC----UGCCAGCAUGAUCUA-- 21

nta-miR167d ----UGAAGC----UGCCAGCAUGAUCUA-- 21

ghr-miR167b ----UGAAGC----UGCCAGCAUGAUCUA-- 21

ghr-miR167a ----UGAAGC----UGCCAGCAUGAUCUA-- 21

ath-miR167a-5p ----UGAAGC----UGCCAGCAUGAUCUA-- 21

ath-miR167b ----UGAAGC----UGCCAGCAUGAUCUA-- 21

tae-miR167b ----UGAAGC----UGACAGCAUGAUCUA-- 21

vvi-miR167c ----UGAAGC----UGCCAGCAUGAUCUC-- 21

cpa-miR167d ----UGAAGC----UGCCAGCAUGAUCUGA- 22

gma-miR167g ----UGAAGC----UGCCAGCAUGAUCUGA- 22

cme-miR167f ----UGAAGC----UGCCAGCAUGAUCUG-- 21

csi-miR167a ----UGAAGC----UGCCAGCAUGAUCUG-- 21

ptc-miR167e ----UGAAGC----UGCCAGCAUGAUCUG-- 21

gma-miR167j ----UGAAGC----UGCCAGCAUGAUCUG-- 21

ptc-miR167h-5p ----UGAAGC----UGCCAACAUGAUCUG-- 21

tae-miR167c-5p ----UGAAGC----UGCCAGCAUGAUCUGC- 22

cme-miR167d ----UGAAGC----UGCCAGCAUGAUCUG-- 21

csi-miR167c ----UGAAGC----UGCCAGCAUGAUCUG-- 21

gma-miR167c ----UGAAGC----UGCCAGCAUGAUCUG-- 21

vvi-miR167a ----UGAAGC----UGCCAGCAUGAUCUG-- 21

osa-miR167h-5p ----UGAAGC----UGCCAGCAUGAUCUG-- 21

osa-miR167e-5p ----UGAAGC----UGCCAGCAUGAUCUG-- 21

osa-miR167d-5p ----UGAAGC----UGCCAGCAUGAUCUG-- 21

osa-miR167g ----UGAAGC----UGCCAGCAUGAUCUG-- 21

osa-miR167f ----UGAAGC----UGCCAGCAUGAUCUG-- 21

osa-miR167i-5p ----UGAAGC----UGCCAGCAUGAUCUG-- 21

osa-miR167j ----UGAAGC----UGCCAGCAUGAUCUG-- 21

sbi-miR167h ----UGAAGC----UGCCAGCAUGAUCUG-- 21

sbi-miR167g ----UGAAGC----UGCCAGCAUGAUCUG-- 21

sbi-miR167d ----UGAAGC----UGCCAGCAUGAUCUG-- 21

sbi-miR167e ----UGAAGC----UGCCAGCAUGAUCUG-- 21

sbi-miR167c ----UGAAGC----UGCCAGCAUGAUCUG-- 21

sbi-miR167f ----UGAAGC----UGCCAGCAUGAUCUG-- 21

sof-miR167a ----UGAAGC----UGCCAGCAUGAUCUG-- 21

sof-miR167b ----UGAAGC----UGCCAGCAUGAUCUG-- 21

mac-miR167c ----UGAAGC----UGCCAGCAUGAUCUG-- 21

ath-miR167d ----UGAAGC----UGCCAGCAUGAUCUGG- 22

nta-miR167c ----UGAAGC----UGCCAGCAUGAUCUGG- 22

nta-miR167b ----UGAAGC----UGCCAGCAUGAUCUGG- 22

nta-miR167a ----UGAAGC----UGCCAGCAUGAUCUGG- 22

mac-miR167d ----UGAAGC----UGCCAGCAUGAUCUGG- 22

mdm-miR167i ----UGAAGC----UGCCAGCAUGAUCUUA- 22

gma-miR167k ----UGAAGC----UGCCAGCCUGAUCUUA- 22

mdm-miR167j ----UGAAGC----UGCCAGCAUGAUCUUA- 22

mdm-miR167h ----UGAAGC----UGCCAGCAUGAUCUUA- 22

cme-miR167c ----UGAAGC----UGCCAGCAUGAUCUU-- 21

cpa-miR167c ----UGAAGC----UGCCAGCAUGAUCUU-- 21

csi-miR167b ----UGAAGC----UGCCAGCAUGAUCUU-- 21

ptc-miR167g-5p ----UGAAGC----UGCCAGCAUGAUCUU-- 21

ptc-miR167f-5p ----UGAAGC----UGCCAGCAUGAUCUU-- 21

gma-miR167e ----UGAAGC----UGCCAGCAUGAUCUU-- 21

gma-miR167f ----UGAAGC----UGCCAGCAUGAUCUU-- 21

ath-miR167c-5p -----UAAGC----UGCCAGCAUGAUCUUG- 21

cme-miR167e ----UCAAGC----UGCCAGCAUGAUCUA-- 21

aqc-miR167 ----UCAAGC----UGCCAGCAUGAUCUA-- 21

gma-miR167h ---AUCAUGC----UGGCAGCUUCAACUGGU 24

gma-miR167i ----UCAUGC----UGGCAGCUUCAACUGGU 23

osa-miR167a-3p ---AUCAUGCA---UGACAGCCUCAUUU--- 22

osa-miR167c-3p --GGUCAUGCU--GCGGCAGCCUCACU---- 23

ptc-miR167h-3p -AGAUCAUGUG-----GCAGUUUCACC---- 21

ptc-miR167f-3p -AGAUCAUGUG-----GCAGUUUCACC---- 21

ptc-miR167g-3p -AGAUCAUGUG-----GCAGUUUCACC---- 21

mdm-miR167a -AGAUCAUCUG-----GCAGUUUCACC---- 21

ath-miR167a-3p --GAUCAUGUUC----GCAGUUUCACC---- 21

osa-miR167i-3p -AGAUCAUGUU-----GCAGCUUCACU---- 21

osa-miR167e-3p -AGAUCAUGUU-----GCAGCUUCACU---- 21

ath-miR167c-3p UAGGUCAUGCUG----GUAGUUUCACC---- 23

osa-miR167h-3p -AGGUCAUGCU-----GUAGUUUCAUC---- 21

osa-miR167d-3p --GAUCAUGCUGU---GCAGUUUCAUC---- 22

* * * *

8. miR169

ath-miR169f-3p -----GCAAGUUGA--CCU-UGG--CUCUG----C--- 21

ptc-miR169n-3p -----GCAAG--CAU-CCU-UGGUUCUC------C--- 20

zma-miR169k-3p ----GGCAGU--CU--CCU-UGG----CUA----G--- 18

zma-miR169j-3p ----GGCAGU--CU--CCU-UGG----CUA----G--- 18

zma-miR169i-3p ----GGCAGU--CU--CCU-UGG----CUA----G--- 18

sbi-miR169d-3p ---GGGCGGU--CA--CCU-UGG----CUA----GC-- 20

zma-miR169o-3p ----GGCAGGU-CU--UCU-UGG----CUA----GC-- 20

zma-miR169q-3p ----GGCAGGC-CUU--CU--GG----CUA----AG-- 19

zma-miR169n-3p ----GGCAGGC-CUU--CU-UGG----CUA----AG-- 20

zma-miR169m-3p ----GGCAU-C-CAU-UCU-UGG----CUA----AG-- 20

gma-miR169h ----GGCGAGA-CAU--CU-UGG----CUC----AUU- 21

osa-miR169r-3p ---UGGCAAGU-CU--CCU-CGG----CUA----CC-- 21

zma-miR169c-3p ----GGCAAGU-CUGUCCU-UGG----CUA----CA-- 22

tae-miR169 ---GGGCAAGU-C-ACCCU-GGG----CUA----CC-- 22

zma-miR169p-3p ----GGCAAGU-CAU-CUG-GGG----CUA----CG-- 21

zma-miR169l-3p ----GGCAAAU-CAUCCCU---G----CUA----CC-- 20

ptc-miR169u-3p ----GGCA-GU-CU--CCUUUGG----CUAU---CC-- 21

zma-miR169f-3p ----GGCAUGU-CU-UCCUU-GG----CUA----CU-- 21

gma-miR169i-3p ------CCGGUGC----C----AU---CCCGUCUCAUA 21

osa-miR169i-3p -----UGAGUCGCU---CU--UAU---CACU---CAUG 22

ath-miR169b-3p ----GGCAAGU-UGU-CCUUCGG----CUACA------ 22

zma-miR169r-3p ----GGCAAGU-UGU-CCU-UGG----CUACA------ 21

ath-miR169a-3p ----GGCAAGU-UGU-CCU-UGG----CUAC------- 20

zma-miR169b-3p ----GGCAAGU-UGUUCU--UGG----CUACA------ 21

zma-miR169a-3p ----GGCAAGU-UGUUCU--UGG----CUACA------ 21

ptc-miR169b-3p ----GGCAGGU-UGUUCUU--GG----CUAC------- 20

gma-miR169l-3p --CGGGCAAGU-UGUUUUU--GG----CUAC------- 22

gma-miR169j-3p UUUCGACGAGU-UGUUCUU--GG----C---------- 21

ath-miR169g-3p -UCCGGCAAGU-UG-ACCU-UGG----CU--------- 21

gma-miR169n-3p UGCCGGCAAGU---UUCUCUUGG----C---------- 21

gma-miR169o --UGAGCCA-GGAU-GGCU-UGC----CGGC------- 22

gma-miR169i-5p --UGAGCCG-GGAU-GGCU-UGC----CGGCA------ 23

gma-miR169r --UGAGCCA-GGAU-GGCU-UGC----CGGC------- 22

vvi-miR169h --UGAGCCAAGGAU-GGCU-UGC----CG--------- 21

vvi-miR169b --UGAGCCAAGGAU-GGCU-UGC----CG--------- 21

ath-miR169g-5p --UGAGCCAAGGAU-GACU-UGC----CG--------- 21

ath-miR169f-5p --UGAGCCAAGGAU-GACU-UGC----CG--------- 21

ath-miR169e --UGAGCCAAGGAU-GACU-UGC----CG--------- 21

ath-miR169d --UGAGCCAAGGAU-GACU-UGC----CG--------- 21

ptc-miR169n-5p --UGAGCCAAGGAU-GACU-UGC----CG--------- 21

gma-miR169p --UGAGCCAAGGAU-GACU-UGC----CG--------- 21

vvi-miR169r --UGAGUCAAGGAU-GACU-UGC----CG--------- 21

vvi-miR169t --CGAGUCAAGGAU-GACU-UGC----CG--------- 21

vvi-miR169u --UGAGUCAAGGAU-GACU-UGC----CG--------- 21

vvi-miR169l ---GAGCCAAGGAU-GACU-UGC----CGU-------- 21

vvi-miR169o ---GAGCCAAGGAU-GACU-UGC----CG-C------- 21

cme-miR169k --UGAGCCAAGGAU-GACU-UGC----CU--------- 21

mdm-miR169b ---UAGCCAAGGAU-GAUU-UGC----CUGC------- 22

osa-miR169r-5p ---UAGCCAAGGAU-GAUU-UGC----CUG-------- 21

sbi-miR169o ---UAGCCAAGGAU-GAUU-UGC----CUG-------- 21

ptc-miR169y ---UAGCCAUGGAU-GAAU-UGC----CUG-------- 21

zma-miR169l-5p ---UAGCCAGGGAU-GAUU-UGC----CUG-------- 21

vvi-miR169e ---UAGCCAAGGAU-GACU-UGC----CUGC------- 22

ath-miR169n ---UAGCCAAGGAU-GACU-UGC----CUG-------- 21

ath-miR169j ---UAGCCAAGGAU-GACU-UGC----CUG-------- 21

ath-miR169k ---UAGCCAAGGAU-GACU-UGC----CUG-------- 21

ath-miR169h ---UAGCCAAGGAU-GACU-UGC----CUG-------- 21

ath-miR169m ---UAGCCAAGGAU-GACU-UGC----CUG-------- 21

ath-miR169i ---UAGCCAAGGAU-GACU-UGC----CUG-------- 21

ath-miR169l ---UAGCCAAGGAU-GACU-UGC----CUG-------- 21

ghr-miR169a ---UAGCCAAGGAU-GACU-UGC----CUG-------- 21

cme-miR169n ---UAGCCAAGGAU-GACU-UGC----CUG-------- 21

cme-miR169e ---UAGCCAAGGAU-GACU-UGC----CUG-------- 21

ptc-miR169k ---UAGCCAAGGAU-GACU-UGC----CUG-------- 21

ptc-miR169l ---UAGCCAAGGAU-GACU-UGC----CUG-------- 21

ptc-miR169m ---UAGCCAAGGAU-GACU-UGC----CUG-------- 21

ptc-miR169j ---UAGCCAAGGAU-GACU-UGC----CUG-------- 21

ptc-miR169i ---UAGCCAAGGAU-GACU-UGC----CUG-------- 21

aqc-miR169b ---UAGCCAAGGAU-GACU-UGC----CUG-------- 21

osa-miR169i-5p.1 ---UAGCCAAGGAU-GACU-UGC----CUG-------- 21

osa-miR169k ---UAGCCAAGGAU-GACU-UGC----CUG-------- 21

osa-miR169l ---UAGCCAAGGAU-GACU-UGC----CUG-------- 21

osa-miR169m ---UAGCCAAGGAU-GACU-UGC----CUG-------- 21

osa-miR169j ---UAGCCAAGGAU-GACU-UGC----CUG-------- 21

osa-miR169h ---UAGCCAAGGAU-GACU-UGC----CUG-------- 21

sbi-miR169f ---UAGCCAAGGAU-GACU-UGC----CUG-------- 21

sbi-miR169l ---UAGCCAAGGAU-GACU-UGC----CUG-------- 21

sbi-miR169g ---UAGCCAAGGAU-GACU-UGC----CUG-------- 21

zma-miR169k-5p ---UAGCCAAGGAU-GACU-UGC----CUG-------- 21

zma-miR169i-5p ---UAGCCAAGGAU-GACU-UGC----CUG-------- 21

zma-miR169j-5p ---UAGCCAAGGAU-GACU-UGC----CUG-------- 21

mac-miR169h ---UAGCCAAGGAU-GACU-UGC----CUG-------- 21

ptc-miR169o ---AAGCCAAGGAU-GACU-UGC----CUG-------- 21

ptc-miR169ag ---AAGCCAAGGGU-GACU-UGC----CUGA------- 22

zma-miR169r-5p ---CAGCCAAGGAU-GACU-UGC----CGG-------- 21

zma-miR169c-5p ---CAGCCAAGGAU-GACU-UGC----CGG-------- 21

sbi-miR169k ---CAGCCAAGGAU-GACU-UGC----CGG-------- 21

sbi-miR169b ---CAGCCAAGGAU-GACU-UGC----CGG-------- 21

osa-miR169c ---CAGCCAAGGAU-GACU-UGC----CGG-------- 21

vvi-miR169c ---CAGCCAAGGAU-GACU-UGC----CGG-------- 21

osa-miR169b ---CAGCCAAGGAU-GACU-UGC----CGG-------- 21

vvi-miR169k ---CAGCCAAGGAU-GACU-UGC----CGG-------- 21

vvi-miR169a ---CAGCCAAGGAU-GACU-UGC----CGG-------- 21

vvi-miR169w ---CAGCCAAGGAU-GACU-UGC----CGG-------- 21

vvi-miR169j ---CAGCCAAGGAU-GACU-UGC----CGG-------- 21

vvi-miR169s ---CAGCCAAGGAU-GACU-UGC----CGG-------- 21

gma-miR169a ---CAGCCAAGGAU-GACU-UGC----CGG-------- 21

gma-miR169f ---CAGCCAAGGAU-GACU-UGC----CGG-------- 21

gma-miR169m ---CAGCCAAGGAU-GACU-UGC----CGG-------- 21

gma-miR169g ---CAGCCAAGGAU-GACU-UGC----CGG-------- 21

aqc-miR169c ---CAGCCAAGGAU-GACU-UGC----CGG-------- 21

ptc-miR169d ---CAGCCAAGGAU-GACU-UGC----CGG-------- 21

ptc-miR169p ---CAGCCAAGGAU-GACU-UGC----CGG-------- 21

ptc-miR169e ---CAGCCAAGGAU-GACU-UGC----CGG-------- 21

ptc-miR169h ---CAGCCAAGGAU-GACU-UGC----CGG-------- 21

ptc-miR169g ---CAGCCAAGGAU-GACU-UGC----CGG-------- 21

ptc-miR169f ---CAGCCAAGGAU-GACU-UGC----CGG-------- 21

mdm-miR169a ---CAGCCAAGGAU-GACU-UGC----CGG-------- 21

cme-miR169h ---CAGCCAAGGAU-GACU-UGC----CGG-------- 21

cme-miR169f ---CAGCCAAGGAU-GACU-UGC----CGG-------- 21

nta-miR169r ---CAGCCAAGGAU-GACU-UGC----CGG-------- 21

nta-miR169q ---CAGCCAAGGAU-GACU-UGC----CGG-------- 21

nta-miR169s ---CAGCCAAGGAU-GACU-UGC----CGG-------- 21

ath-miR169c ---CAGCCAAGGAU-GACU-UGC----CGG-------- 21

ath-miR169b-5p ---CAGCCAAGGAU-GACU-UGC----CGG-------- 21

ath-miR169a-5p ---CAGCCAAGGAU-GACU-UGC----CGA-------- 21

nta-miR169b ---CAGCCAAGGAU-GACU-UGC----CGA-------- 21

nta-miR169c ---CAGCCAAGGAU-GACU-UGC----CGA-------- 21

nta-miR169f ---CAGCCAAGGAU-GACU-UGC----CGA-------- 21

nta-miR169h ---CAGCCAAGGAU-GACU-UGC----CGA-------- 21

nta-miR169a ---CAGCCAAGGAU-GACU-UGC----CGA-------- 21

nta-miR169k ---CAGCCAAGGAU-GACU-UGC----CGA-------- 21

nta-miR169l ---CAGCCAAGGAU-GACU-UGC----CGA-------- 21

nta-miR169i ---CAGCCAAGGAU-GACU-UGC----CGA-------- 21

nta-miR169d ---CAGCCAAGGAU-GACU-UGC----CGA-------- 21

nta-miR169e ---CAGCCAAGGAU-GACU-UGC----CGA-------- 21

nta-miR169g ---CAGCCAAGGAU-GACU-UGC----CGA-------- 21

nta-miR169j ---CAGCCAAGGAU-GACU-UGC----CGA-------- 21

nta-miR169o ---CAGCCAAGGAU-GACU-UGC----CGA-------- 21

nta-miR169p ---CAGCCAAGGAU-GACU-UGC----CGA-------- 21

nta-miR169m ---CAGCCAAGGAU-GACU-UGC----CGA-------- 21

ptc-miR169b-5p ---CAGCCAAGGAU-GACU-UGC----CGA-------- 21

ptc-miR169c ---CAGCCAAGGAU-GACU-UGC----CGA-------- 21

ptc-miR169a ---CAGCCAAGGAU-GACU-UGC----CGA-------- 21

gma-miR169b ---CAGCCAAGGAU-GACU-UGC----CGA-------- 21

vvi-miR169g ---CAGCCAAGGAU-GACU-UGC----CGA-------- 21

vvi-miR169f ---CAGCCAAGGAU-GACU-UGC----CGA-------- 21

osa-miR169a ---CAGCCAAGGAU-GACU-UGC----CGA-------- 21

sbi-miR169a ---CAGCCAAGGAU-GACU-UGC----CGA-------- 21

zma-miR169b-5p ---CAGCCAAGGAU-GACU-UGC----CGA-------- 21

zma-miR169a-5p ---CAGCCAAGGAU-GACU-UGC----CGA-------- 21

gma-miR169c ---AAGCCAAGGAU-GACU-UGC----CGA-------- 21

ptc-miR169s --UCAGCCAAGGAU-GACU-UGC----CG--------- 21

gma-miR169u ---CAGCCAAGGAU-GACU-UGC----CGU-------- 21

gma-miR169v ---CAGCCAAGGAU-GACU-UGC----C---------- 19

ghr-miR169b ---CAGCCAAGGAU-GAUU-UGC----CGG-------- 21

gma-miR169n-5p ---CAGCCAAGGGU-GAUU-UGC----CGG-------- 21

ptc-miR169z ---CAGCCAAGAAU-GAUU-UGC----CGG-------- 21

vvi-miR169d ---CAGCCAAGAAU-GAUU-UGC----CGG-------- 21

cme-miR169t --UGAGCCAAGAAU-GACU-UGC----CGGC------- 23

ptc-miR169aa ---GAGCCAAGAAU-GACU-UGU----CGG-------- 21

cme-miR169r ---GAGCCAAGAAU-GACU-UGC----CGG-------- 21

ptc-miR169t ---GAGCCAAGAAU-GACU-UGC----CGG-------- 21

csi-miR169 ---GAGCCAAGAAU-GACU-UGC----CGA-------- 21

gma-miR169j-5p ---UAGCCAAGAAU-GACU-UGC----CGG-------- 21

gma-miR169k ---CAGCCAAGAAU-GACU-UGC----CGG-------- 21

gma-miR169l-5p ---CAGCCAAGAAU-GACU-UGC----CGG-------- 21

cpa-miR169 ---CAGCCAAGAAU-GACU-UGC----CG--------- 20

gma-miR169e ----AGCCAAGGAU-GACU-UGC----CGG-------- 20

sbi-miR169e ---UAGCCAAGGAU-GACU-UGC----CGG-------- 21

zma-miR169p-5p ---UAGCCAAGGAU-GACU-UGC----CGG-------- 21

osa-miR169p ---UAGCCAAGGACAAACU-UGC----CGG-------- 22

osa-miR169e ---UAGCCAAGGAU-GACU-UGC----CGG-------- 21

sbi-miR169j ---UAGCCAAGGAU-GACU-UGC----CGG-------- 21

gma-miR169d --UGAGCCAAGGAU-GACU-UGC----CGGU------- 23

vvi-miR169m ---GAGCCAAGGAU-GACU-UGC----CGG-------- 21

vvi-miR169p ---GAGCCAAGGAU-GACU-UGC----CGG-------- 21

vvi-miR169n ---GAGCCAAGGAU-GACU-UGC----CGG-------- 21

vvi-miR169q ---GAGCCAAGGAU-GACU-UGC----CGG-------- 21

cme-miR169g ---AAGCCAAGGAU-GAAU-UGC----CGG-------- 21

vvi-miR169v ---AAGCCAAGGAU-GAAU-UGC----CGG-------- 21

osa-miR169d ---UAGCCAAGGAU-GAAU-UGC----CGG-------- 21

gma-miR169s ---AAGCCAAGGAU-GACU-UGC----CGG-------- 21

mdm-miR169d ---UAGCCAAGGAU-GACU-UGCC---CG--------- 21

mdm-miR169c ---UAGCCAAGGAU-GACU-UGCC---CG--------- 21

ptc-miR169x ---UAGCCAAGGAU-GACU-UGCU---CG--------- 21

cme-miR169i ---UAGCCAAAAAU-GACU-UGC----CUGC------- 22

cme-miR169m ---UAGCCAAAAAU-GACU-UGC----CUGC------- 22

cme-miR169l ---UAGCCAAAAAU-GACU-UGC----CUGC------- 22

cme-miR169j ---UAGCCAAAAAU-GACU-UGC----CUGC------- 22

cme-miR169b ---UAGCCAAAAAU-GACU-UGC----CUG-------- 21

cme-miR169a ---UAGCCAAAAAU-GACU-UGC----CUG-------- 21

cme-miR169c ---UAGCCAAAGAU-GACU-UGC----CUG-------- 21

cme-miR169d ---UAGCCAAAGAU-GACU-UGC----CUG-------- 21

cme-miR169o ---UAGCCAAAGAU-GACU-UGC----CUG-------- 21

cme-miR169p --UGAGCCAAAGAU-GACU-UGC----CU--------- 21

cme-miR169q --UGAGCCAAAGAU-GACU-UGC----CU--------- 21

cme-miR169s --UGAGCCAAAGAU-GACU-UGC----CU--------- 21

ssl-miR169 ---UAGCCAAGGAU-GACU-UGC----CUA-------- 21

vvi-miR169y ---UAGCGAAGGAU-GACU-UGC----CUA-------- 21

ptc-miR169r ---UAGCCAAGGAU-GACU-UGC----CUA-------- 21

vvi-miR169x ---UAGCCAAGGAU-GACU-UGC----CUA-------- 21

osa-miR169f.1 ---UAGCCAAGGAU-GACU-UGC----CUA-------- 21

sbi-miR169c ---UAGCCAAGGAU-GACU-UGC----CUA-------- 21

sbi-miR169h ---UAGCCAAGGAU-GACU-UGC----CUA-------- 21

zma-miR169f-5p ---UAGCCAAGGAU-GACU-UGC----CUA-------- 21

osa-miR169o ---UAGCCAAGAAU-GACU-UGC----CUA-------- 21

osa-miR169n ---UAGCCAAGAAU-GACU-UGC----CUA-------- 21

sbi-miR169i ---UAGCCAAGAAU-GACU-UGC----CUA-------- 21

zma-miR169o-5p ---UAGCCAAGAAU-GACU-UGC----CUA-------- 21

sbi-miR169p ---UAGCCAAGAAU-GGCU-UGC----CUA-------- 21

sbi-miR169q ---UAGCCAAGAAU-GGCU-UGC----CUA-------- 21

zma-miR169q-5p ---UAGCCAAGAAU-GGCU-UGC----CUA-------- 21

zma-miR169m-5p ---UAGCCAAGAAU-GGCU-UGC----CUA-------- 21

zma-miR169n-5p ---UAGCCAAGAAU-GGCU-UGC----CUA-------- 21

gma-miR169t ---UAGCCAAGGAUGGACU-UGC----CUA-------- 22

aqc-miR169a ---UAGCCAAGGAU-GACU-UGC----CUA-------- 21

osa-miR169g ---UAGCCAAGGAU-GACU-UGC----CUA-------- 21

sbi-miR169n ---UAGCCAAGGAU-GACU-UGC----CUA-------- 21

sbi-miR169m ---UAGCCAAGGAU-GACU-UGC----CUA-------- 21

zma-miR169h ---UAGCCAAGGAU-GACU-UGC----CUA-------- 21

zma-miR169g ---UAGCCAAGGAU-GACU-UGC----CUA-------- 21

ptc-miR169ac ---UAGCCAAGGA-CGACU-UGC----CCA-------- 21

ptc-miR169ad ---UAGCCAAGGA-CGACU-UGC----CCA-------- 21

ptc-miR169af ---UAGCCAAGGA-CGACU-UGC----CCA-------- 21

ptc-miR169ae ---UAGCCAAGGA-CGACU-UGC----CCA-------- 21

ptc-miR169u-5p ---UAGCCAAGGA-CGACU-UGC----CUA-------- 21

ptc-miR169q ---UAGCCAAGGA-CGACU-UGC----CUG-------- 21

ptc-miR169w ---UAGCCAAGGAU-GACU-UGC----CCA-------- 21

ptc-miR169v ---UAGCCAAGGAU-GACU-UGC----CCA-------- 21

ptc-miR169ab ---CAGCCAAGGAA-GACU-UGC----CC--------- 20

nta-miR169t ---UAGCCAAGGAU-GACU-UGC----CUU-------- 21

sbi-miR169d-5p ---UAGCCAAGGAU-GACU-UGC----CU--------- 20

vvi-miR169i ---GAGCCAAGGAU-GACU-GGC----CGU-------- 21

zma-miR169d ---UAGCCAAGGA--GACU--GC----CUAUG------ 21

zma-miR169e ---UAGCCAAGGA--GACU--GC----CUACG------ 21

osa-miR169q ---UAGCCAAGGA--GACU--GC----CCAUG------ 21

mdm-miR169f ----UGAAGAGAAG-AGCGUUGUU---UGG-------- 22

mdm-miR169e ----UGAAGAGAAG-AGCGUUGUU---UGG-------- 22

osa-miR169f.2 ----UGAGGACAAG-AGCU--GAU--UCGG-------- 21

osa-miR169i-5p.2 ----UGGUGAUAAG-GGUGUAGCU---CUG-------- 22

9. miR172

ath-miR172b-3p --AGAAUCUUGAUGAUGCUGCAU--- 21

nta-miR172b --AGAAUCAUGAUGAUGCUGCAU--- 21

ath-miR172a --AGAAUCUUGAUGAUGCUGCAU--- 21

nta-miR172g --AGAAUCUUGAUGAUGCUGCAU--- 21

nta-miR172i --AGAAUCUUGAUGAUGCUGCAU--- 21

nta-miR172e --AGAAUCUUGAUGAUGCUGCAU--- 21

nta-miR172h --AGAAUCUUGAUGAUGCUGCAU--- 21

nta-miR172f --AGAAUCUUGAUGAUGCUGCAU--- 21

nta-miR172c --AGAAUCUUGAUGAUGCUGCAU--- 21

nta-miR172d --AGAAUCUUGAUGAUGCUGCAU--- 21

cme-miR172b --AGAAUCUUGAUGAUGCUGCAU--- 21

cme-miR172c --AGAAUCUUGAUGAUGCUGCAU--- 21

mdm-miR172h --AGAAUCUUGAUGAUGCUGCAU--- 21

mdm-miR172d --AGAAUCUUGAUGAUGCUGCAU--- 21

mdm-miR172e --AGAAUCUUGAUGAUGCUGCAU--- 21

mdm-miR172g --AGAAUCUUGAUGAUGCUGCAU--- 21

mdm-miR172f --AGAAUCUUGAUGAUGCUGCAU--- 21

ssl-miR172 --AGAAUCUUGAUGAUGCUGCAU--- 21

ptc-miR172b-3p --AGAAUCUUGAUGAUGCUGCAU--- 21

ptc-miR172f --AGAAUCUUGAUGAUGCUGCAU--- 21

ptc-miR172c --AGAAUCUUGAUGAUGCUGCAU--- 21

ptc-miR172a --AGAAUCUUGAUGAUGCUGCAU--- 21

aqc-miR172a --AGAAUCUUGAUGAUGCUGCAU--- 21

gma-miR172a --AGAAUCUUGAUGAUGCUGCAU--- 21

gma-miR172h-3p --AGAAUCUUGAUGAUGCUGCAU--- 21

gma-miR172b-3p --AGAAUCUUGAUGAUGCUGCAU--- 21

osa-miR172d-3p --AGAAUCUUGAUGAUGCUGCAU--- 21

osa-miR172a --AGAAUCUUGAUGAUGCUGCAU--- 21

vvi-miR172d UGAGAAUCUUGAUGAUGCUGCAU--- 23

zma-miR172c-3p --AGAAUCUUGAUGAUGCUGCA---- 20

zma-miR172a --AGAAUCUUGAUGAUGCUGCA---- 20

zma-miR172b-3p --AGAAUCUUGAUGAUGCUGCA---- 20

zma-miR172d-3p --AGAAUCUUGAUGAUGCUGCA---- 20

sbi-miR172d --AGAAUCUUGAUGAUGCUGCA---- 20

sbi-miR172a --AGAAUCUUGAUGAUGCUGCA---- 20

sbi-miR172c --AGAAUCUUGAUGAUGCUGCA---- 20

gma-miR172f --AGAAUCUUGAUGAUGCUGCA---- 20

csi-miR172a-3p --AGAAUCUUGAUGAUGCUGCA---- 20

mdm-miR172b --AGAAUCUUGAUGAUGCUGCA---- 20

ath-miR172e-3p --GGAAUCUUGAUGAUGCUGCAU--- 21

nta-miR172j --GGAAUCUUGAUGAUGCUGCAU--- 21

cme-miR172d --GGAAUCUUGAUGAUGCUGCAU--- 21

mdm-miR172k --GGAAUCUUGAUGAUGCUGCAU--- 21

mdm-miR172j --GGAAUCUUGAUGAUGCUGCAU--- 21

mdm-miR172i --GGAAUCUUGAUGAUGCUGCAU--- 21

ptc-miR172e --GGAAUCUUGAUGAUGCUGCAU--- 21

ptc-miR172d --GGAAUCUUGAUGAUGCUGCAU--- 21

aqc-miR172b --GGAAUCUUGAUGAUGCUGCAU--- 21

gma-miR172i-3p --GGAAUCUUGAUGAUGCUGCAU--- 21

gma-miR172l --GGAAUCUUGAUGAUGCUGCAU--- 21

osa-miR172b --GGAAUCUUGAUGAUGCUGCAU--- 21

zma-miR172e --GGAAUCUUGAUGAUGCUGCAU--- 21

gma-miR172d --GGAAUCUUGAUGAUGCUGCAGCAG 24

gma-miR172e --GGAAUCUUGAUGAUGCUGCAGCAG 24

cme-miR172a --GGAAUCUUGAUGAUGCUGCAG--- 21

mdm-miR172l --GGAAUCUUGAUGAUGCUGCAG--- 21

ptc-miR172h-3p --GGAAUCUUGAUGAUGCUGCAG--- 21

vvi-miR172c --GGAAUCUUGAUGAUGCUGCAG--- 21

csi-miR172c -UGGAAUCUUGAUGAUGCUGCAG--- 22

ptc-miR172g-3p --GGAAUCUUGAUGAUGCUGCAG--- 21

gma-miR172c --GGAAUCUUGAUGAUGCUGCAG--- 21

cpa-miR172a -GGGAAUCUUGAUGAUGCUGCA---- 21

cpa-miR172b -GGGAAUCUUGAUGAUGCUGCA---- 21

sbi-miR172b --GGAAUCUUGAUGAUGCUGCA---- 20

osa-miR172c --UGAAUCUUGAUGAUGCUGCAC--- 21

sbi-miR172e --UGAAUCUUGAUGAUGCUGCAC--- 21

cme-miR172f --UGAAUCUUGAUGAUGCCGCAC--- 21

mac-miR172b --UGAAUCUUAAUGAUGCUACA---- 20

mac-miR172c --UGAAUCUUAAUGAUGCUACAC--- 21

vvi-miR172b --UGAAUCUUGAUGAUGCUACAC--- 21

vvi-miR172a --UGAAUCUUGAUGAUGCUACAU--- 21

gma-miR172k --UGAAUCUUGAUGAUGCUGCAU--- 21

mdm-miR172c --AGAAUCUUGAUGAUGCUGCA---- 20

mdm-miR172a --AGAAUCUUGAUGAUGCUGCA---- 20

ath-miR172c --AGAAUCUUGAUGAUGCUGCAG--- 21

ath-miR172d-3p --AGAAUCUUGAUGAUGCUGCAG--- 21

nta-miR172a --AGAAUCUUGAUGAUGCUGCAG--- 21

cme-miR172e --AGAAUCUUGAUGAUGCUGCAG--- 21

mdm-miR172n --AGAAUCUUGAUGAUGCUGCAG--- 21

mdm-miR172m --AGAAUCUUGAUGAUGCUGCAG--- 21

mdm-miR172o --AGAAUCUUGAUGAUGCUGCAG--- 21

ptc-miR172i --AGAAUCCUGAUGAUGCUGCAA--- 21

sbi-miR172f --AGAAUCCUGAUGAUGCUGCAC--- 21

ghr-miR172 --AGAAUCCUGAUGAUGCUGCAG--- 21

csi-miR172b --AGAAUCUUGAUGAUGCGGCAA--- 21

ath-miR172e-5p GCAGCACCAUUAAGAU-UCAC----- 20

ath-miR172b-5p GCAGCACCAUUAAGAU-UCAC----- 20

gma-miR172j GCAGCAGCAUCAAGAU-UCACA---- 21

zma-miR172c-5p -CAGCACCACCAAGAU-UCACA---- 20

gma-miR172i-5p GCAGCAGCAUCAAGAU-UCACA---- 21

gma-miR172h-5p GCAGCAGCAUCAAGAU-UCACA---- 21

gma-miR172g GCAGCACCAUCAAGAU-UCAC----- 20

osa-miR172d-5p GCAGCACCAUCAAGAU-UCAC----- 20

zma-miR172d-5p -CAGCACCAUCAAGAU-UCACA---- 20

zma-miR172b-5p -CAGCACCAUCAAGAU-UCACA---- 20

ptc-miR172h-5p GGAGCACCAUCAAGAU-UCACA---- 21

csi-miR172a-5p GCAGCGUCCUCAAGAU-UCACA---- 21

ptc-miR172b-5p GGAGCAUCAUCAAGAU-UCACA---- 21

ptc-miR172g-5p GGAGCAUCAUCAAGAU-UCACA---- 21

gma-miR172b-5p GUAGCAUCAUCAAGAU-UCAC----- 20

ath-miR172d-5p GCAACAUCUUCAAGAU-UCAGA---- 21

* * ***

10. miR319

csi-miR319 -UUUGGACUGAAG-GG-AGCUCCU--- 21

vvi-miR319e -UUUGGACUGAAG-GG-AGCUCCU--- 21

cme-miR319c --UUGGACUGAAG-GG-AGCUCCU--- 20

cme-miR319d --UUGGACUGAAG-GG-AGCUCCU--- 20

ptc-miR319f --UUGGACUGAAG-GG-AGCUCCU--- 20

ptc-miR319g --UUGGACUGAAG-GG-AGCUCCU--- 20

ptc-miR319h --UUGGACUGAAG-GG-AGCUCCU--- 20

gma-miR319g --UUGGACUGAAG-GG-AGCUCCUUC- 22

gma-miR319l --UUGGACUGAAG-GG-AGCUCCUUC- 22

ptc-miR319e --UUGGACUGAAG-GG-AGCUCCU--- 20

gma-miR319c --UUGGACUGAAA-GG-AGCUCCU--- 20

ath-miR319a --UUGGACUGAAG-GG-AGCUCCCU-- 21

ath-miR319b --UUGGACUGAAG-GG-AGCUCCCU-- 21

nta-miR319b --UUGGACUGAAG-GG-AGCUCCCU-- 21

nta-miR319a --UUGGACUGAAG-GG-AGCUCCCU-- 21

tae-miR319 --UUGGACUGAAG-GG-AGCUCCCU-- 21

mac-miR319m --UUGGACUGAAG-GG-AGCUCCCU-- 21

vvi-miR319b --UUGGACUGAAG-GG-AGCUCCCU-- 21

vvi-miR319f --UUGGACUGAAG-GG-AGCUCCCU-- 21

vvi-miR319c --UUGGACUGAAG-GG-AGCUCCCU-- 21

gma-miR319j --UUGGACUGAAG-GG-AGCUCCCU-- 21

gma-miR319k --UUGGACUGAAG-GG-AGCUCCCU-- 21

gma-miR319m --UUGGACUGAAG-GG-AGCUCCCU-- 21

gma-miR319h --UUGGACUGAAG-GG-AGCUCCCU-- 21

hbr-miR319 --UUGGACUGAAG-GG-AGCUCCCU-- 21

aqc-miR319 --UUGGACUGAAG-GG-AGCUCCCU-- 21

mdm-miR319b --UUGGACUGAAG-GG-AGCUCCCU-- 21

mdm-miR319a --UUGGACUGAAG-GG-AGCUCCCU-- 21

mac-miR319c --UUGGACUGAAG-GG-AGCUCCC--- 20

gma-miR319b --UUGGACUGAAG-GG-AGCUCCC--- 20

gma-miR319a --UUGGACUGAAG-GG-AGCUCCC--- 20

gma-miR319e --UUGGACUGAAG-GG-AGCUCCC--- 20

ptc-miR319b --UUGGACUGAAG-GG-AGCUCCC--- 20

ptc-miR319c --UUGGACUGAAG-GG-AGCUCCC--- 20

ptc-miR319a --UUGGACUGAAG-GG-AGCUCCC--- 20

ptc-miR319i --UUGGGCUGAAG-GG-AGCUCCC--- 20

cme-miR319b --UUGGACUGAAG-GG-AGCUCCC--- 20

vvi-miR319g --UUGGACUGAAG-GG-AGCUCCCA-- 21

cme-miR319a --UUGGACUGAAG-GG-AGCUCCC--- 20

ptc-miR319d --UUGGACUGAAG-GG-AGCUCCC--- 20

osa-miR319a-3p.2-3p --UUGGACUGAAG-GG-UGCUCCC--- 20

osa-miR319b --UUGGACUGAAG-GG-UGCUCCC--- 20

zma-miR319c-3p --UUGGACUGAAG-GG-UGCUCCC--- 20

zma-miR319d-3p --UUGGACUGAAG-GG-UGCUCCC--- 20

zma-miR319a-3p --UUGGACUGAAG-GG-UGCUCCC--- 20

zma-miR319b-3p --UUGGACUGAAG-GG-UGCUCCC--- 20

sbi-miR319a --UUGGACUGAAG-GG-UGCUCCC--- 20

sbi-miR319b --UUGGACUGAAG-GG-UGCUCCC--- 20

cpa-miR319 -AUUGGACUGAAG-GG-AGCUCC---- 20

gma-miR319p UUUUGGACUGAAG-GG-AGCUCC---- 21

gma-miR319f --UUGGACUGAAG-GG-GCCUCUU--- 20

gma-miR319n -UUUGGACCGAAG-GG-AGCCCCU--- 21

gma-miR319o ---UGGACUGAAG-GGGAGCUCCUUC- 22

gma-miR319d ---UGGACUGAAG-GGGAGCUCCUUC- 22

gma-miR319q ---UGGACUGAAG-GG-AGCUCCUUC- 21

gma-miR319i --UUGGACUGAAG-GGGAGCUCCUUC- 23

osa-miR319a-3p -ACUGGA-UGACGCGGGAGCUAA---- 21

mdm-miR319c -AUCCAAC-GAAGCAGGAGCUGA---- 21

zma-miR319c-5p ----GAGCUCUCU--UCAGUCCACUC- 20

zma-miR319a-5p ----GAGCUCUCU--UCAGUCCACUC- 20

zma-miR319b-5p ---AGAGCGUCCU--UCAGUCCACUC- 21

zma-miR319d-5p ---AGAGCGUCCU--UCAGUCCACUC- 21

osa-miR319a-5p -----AGCUGCCGAAUCA-UCCAUUCA 21

11. miR397

ath-miR397a UCAUUGAGUGCAGCGUUGAUG--- 21

ath-miR397b UCAUUGAGUGCAUCGUUGAUG--- 21

cme-miR397 UCAUUGAGUGCAGCGUUGAUG--- 21

csi-miR397 UCAUUGAGUGCAGCGUUGAUG--- 21

ssl-miR397 UCAUUGAGUGCAGCGUUGAUG--- 21

mbg-miR397a UCAUUGAGUGCAGCGUUGAUG--- 21

nta-miR397 --AUUGAGUGCAGCGUUGAUGU-- 20

mdm-miR397a ---UUGAGUGCAGCGUUGAUGAAA 21

mdm-miR397b ---UUGAGUGCAGCGUUGAUGAAA 21

********* ********

12. miR399

ath-miR399b -UG--CCAAAGGAGAGUUGCCCU--G---- 21

ath-miR399c-3p -UG--CCAAAGGAGAGUUGCCCU--G---- 21

mdm-miR399i -UG--CCAAAGGAGAGUUGCCCU--G---- 21

mdm-miR399j -UG--CCAAAGGAGAGUUGCCCU--G---- 21

csi-miR399d -UG--CCAAAGGAGAGUUGCCCU--G---- 21

gma-miR399a -UG--CCAAAGGAGAGUUGCCCU--G---- 21

gma-miR399h -UG--CCAAAGGAGAGUUGCCCU--G---- 21

gma-miR399b -UG--CCAAAGGAGAGUUGCCCU--G---- 21

gma-miR399c -UG--CCAAAGGAGAGUUGCCCU--G---- 21

vvi-miR399b -UG--CCAAAGGAGAGUUGCCCU--G---- 21

vvi-miR399c -UG--CCAAAGGAGAGUUGCCCU--G---- 21

osa-miR399d -UG--CCAAAGGAGAGUUGCCCU--G---- 21

sbi-miR399d -UG--CCAAAGGAGAGUUGCCCU--G---- 21

sbi-miR399i -UG--CCAAAGGAGAGUUGCCCU--G---- 21

zma-miR399j-3p -UG--CCAAAGGAGAGUUGCCCU--G---- 21

zma-miR399i-3p -UG--CCAAAGGAGAGUUGCCCU--G---- 21

zma-miR399e-3p -UG--CCAAAGGAGAGUUGCCCU--G---- 21

zma-miR399h-3p -UG--CCAAAGGAGAAUUGCCCU--G---- 21

mbg-miR399a -UG--CCAAAGGAGAAUUGCCCU--G---- 21

zma-miR399c-3p -UG--CCAAAGGAGAAUUGCCCU--G---- 21

zma-miR399a-3p -UG--CCAAAGGAGAAUUGCCCU--G---- 21

sbi-miR399j -UG--CCAAAGGAGAAUUGCCCU--G---- 21

sbi-miR399a -UG--CCAAAGGAGAAUUGCCCU--G---- 21

sbi-miR399h -UG--CCAAAGGAGAAUUGCCCU--G---- 21

sbi-miR399c -UG--CCAAAGGAGAAUUGCCCU--G---- 21

osa-miR399c -UG--CCAAAGGAGAAUUGCCCU--G---- 21

osa-miR399a -UG--CCAAAGGAGAAUUGCCCU--G---- 21

osa-miR399b -UG--CCAAAGGAGAAUUGCCCU--G---- 21

vvi-miR399a -UG--CCAAAGGAGAAUUGCCCU--G---- 21

vvi-miR399h -UG--CCAAAGGAGAAUUGCCCU--G---- 21

gma-miR399i -UG--CCAAAGGAGAAUUGCCCU--G---- 21

ptc-miR399f -UG--CCAAAGGAGAAUUGCCCU--G---- 21

ptc-miR399g -UG--CCAAAGGAGAAUUGCCCU--G---- 21

csi-miR399e -UG--CCAAAGGAGAAUUGCCCU--G---- 21

csi-miR399c -UG--CCAAAGGAGAAUUGCCCU--G---- 21

mdm-miR399c -UG--CCAAAGGAGAAUUGCCCU--G---- 21

mdm-miR399b -UG--CCAAAGGAGAAUUGCCCU--G---- 21

mdm-miR399a -UG--CCAAAGGAGAAUUGCCCU--G---- 21

cme-miR399f -UG--CCAAAGGAGAAUUGCAC-------- 19

tae-miR399 -UG--CCAAAGGAGAAUUGCCC-------- 19

osa-miR399k -UG--CCAAAGGAAAUUUGCCCC--G---- 21

sbi-miR399g -UG--CCAAAGGAAAUUUGCCCC--G---- 21

zma-miR399f-3p -UG--CCAAAGGAAAUUUGCCCC--G---- 21

ptc-miR399d -UG--CCAAAGAAGAUUUGCCCC--G---- 21

ath-miR399d -UG--CCAAAGGAGAUUUGCCCC--G---- 21

ghr-miR399e -UG--CCAAAGGAGAUUUGCCCC--G---- 21

cme-miR399a -UG--CCAAAGGAGAUUUGCCCC--G---- 21

ptc-miR399a -UG--CCAAAGGAGAUUUGCCCC--G---- 21

vvi-miR399g -UG--CCAAAGGAGAUUUGCCCC--U---- 21

ath-miR399e -UG--CCAAAGGAGAUUUGCCUC--G---- 21

vvi-miR399d -UG--CCAAAGGAGAUUUGC-UC--GU--- 21

mdm-miR399f -UG--CCAAAGGAGAUUUGC-UC--GG--- 21

mdm-miR399e -UG--CCAAAGGAGAUUUGC-UC--GG--- 21

mdm-miR399g -UG--CCAAAGGAGAUUUGC-UC--GG--- 21

mdm-miR399h -UG--CCAAAGGAGAUUUGC-UC--GG--- 21

ptc-miR399c -UG--CCAAAGGAGAUUUGC-UC--AC--- 21

gma-miR399f -UG--CCAAAGGAGAUUUGCCCA--G---- 21

gma-miR399g -UG--CCAAAGGAGAUUUGCCCA--G---- 21

gma-miR399d -UG--CCAAAGGAGAUUUGCCCA--G---- 21

gma-miR399e -UG--CCAAAGGAGAUUUGCCCA--G---- 21

osa-miR399g -UG--CCAAAGGAGAUUUGCCCA--G---- 21

osa-miR399e -UG--CCAAAGGAGAUUUGCCCA--G---- 21

osa-miR399f -UG--CCAAAGGAGAUUUGCCCA--G---- 21

sbi-miR399f -UG--CCAAAGGAGAUUUGCCCA--G---- 21

sbi-miR399e -UG--CCAAAGGAGAUUUGCCCA--G---- 21

ath-miR399f -UG--CCAAAGGAGAUUUGCCCG--G---- 21

sbi-miR399k -UG--CCAAAGGGGAUUUGCCCG--G---- 21

cme-miR399c -UG--CCAAAGGAGAUUUGCCCG--G---- 21

csi-miR399a -UG--CCAAAGGAGAUUUGCCCG--G---- 21

ptc-miR399b -UG--CCAAAGGAGAUUUGCCCG--G---- 21

vvi-miR399e -UG--CCAAAGGAGAUUUGCCCG--G---- 21

ghr-miR399a -CG--CCAAUGGAGAUUUGUCCG--G---- 21

ghr-miR399b -CG--CCAAUGGAGAUUUGUCCG--G---- 21

ptc-miR399j -UG--CCAAAGGAGAUUUGUCCG--G---- 21

osa-miR399h -UG--CCAAAGGAGACUUGCCCA--G---- 21

ath-miR399a -UG--CCAAAGGAGAUUUGCCCU--G---- 21

ghr-miR399d -UG--CCAAAGGAGAUUUGCCCU--G---- 21

ptc-miR399h -UG--CCAAAGGAGAGUUUCCCU--G---- 21

ghr-miR399c -UG--CCAAAGGAGAGUUGGCCU--U---- 21

cme-miR399d -UG--CCAAAGGAGAGUUGCCCU--U---- 21

cme-miR399e -UG--CCAAAGGAGAGUUGCCCU--U---- 21

cme-miR399b -UG--CCAAAGGAGAGUUGCCCU--A---- 21

mdm-miR399d -UG--CCAAAGGAGAGUUGCCCU--A---- 21

csi-miR399b -UG--CCAAAGGAGAGUUGCCCU--A---- 21

ptc-miR399i -UG--CCAAAGGAGAGUUGCCCU--A---- 21

aqc-miR399 -UG--CCAAAGGAGAGUUGCCCU--A---- 21

osa-miR399j -UG--CCAAAGGAGAGUUGCCCU--A---- 21

ptc-miR399e -CG--CCAAAGGAGAGUUGCCCU--C---- 21

vvi-miR399i -CG--CCAAAGGAGAGUUGCCCU--G---- 21

nta-miR399a -CG--CCAAAGGAGAGCUGCCCU--G---- 21

nta-miR399c -CG--CCAAAGGAGAGCUGCCCU--G---- 21

nta-miR399g -CG--CCAAAGGAGAGCUGCCCU--G---- 21

nta-miR399d -CG--CCAAAGGAGAGCUGCCCU--G---- 21

nta-miR399e -CG--CCAAAGGAGAGCUGCCCU--G---- 21

nta-miR399f -CG--CCAAAGGAGAGCUGCCCU--G---- 21

nta-miR399b -CG--CCAAAGGAGAGCUGCCCU--G---- 21

osa-miR399i -UG--CCAAAGGAGAGCUGCCCU--G---- 21

sbi-miR399b -UG--CCAAAGGAGAGCUGCCCU--G---- 21

zma-miR399d-3p -UG--CCAAAGGAGAGCUGCCCU--G---- 21

zma-miR399b-3p -UG--CCAAAGGAGAGCUGUCCU--G---- 21

vvi-miR399f -UG--CCGAAGGAGAUUUGUCCU--G---- 21

ath-miR319c UUGGACUGAAGG-GAGCUCCUU-------- 21

ath-miR399c-5p -------GGGCAUC-UUUCUAUU-GGCAGG 21

zma-miR399e-5p -------GGGCUUC-UCUUUCUU-GGCAGG 21

cme-miR399g ------AGGGCUUC-UCUCCAUU-GGCAGG 22

zma-miR399f-5p -------GGGCAACUUCUCCUUU-GGCAGA 22

zma-miR399c-5p -------GGGUA-CGUCUCCUUU-GGCACA 21

zma-miR399j-5p -------AGGCAGC-UCUCCUCU-GGCAGG 21

zma-miR399i-5p -------GUGCGGC-UCUCCUCU-GGCAUG 21

zma-miR399d-5p -------GUGUGGC-UCUCCUCU-GGCAUG 21

zma-miR399b-5p -------GUGCAGC-UCUCCUCU-GGCAUG 21

zma-miR399a-5p -------GUGCGGU-UCUCCUCU-GGCACG 21

zma-miR399h-5p -------GUGCAGU-UCUCCUCU-GGCACG 21

mbg-miR399a* -------GUGCAGU-UCUCCUCUUGGCAA- 21

*

13. miR4995

gma-miR4995 AGGCAGUGGCUUGGUUAAGGG 21

mac-miR4995 AGGCAGUGGCUUGGUUAAGGG 21

*********************

14. miR5538

osa-miR5538 ACUGAACUCAAUCACUUGCUGC 22

mac-miR5538 ACUGAACUCAAUCACUUGCUGC 22

**********************
